# Haemoglobin thresholds to define anaemia in a national sample of healthy children and adolescents aged 1–19 years in India: a population-based study

**DOI:** 10.1016/S2214-109X(21)00077-2

**Published:** 2021-04-16

**Authors:** Harshpal Singh Sachdev, Akash Porwal, Rajib Acharya, Sana Ashraf, Sowmya Ramesh, Nizamuddin Khan, Umesh Kapil, Anura V Kurpad, Avina Sarna

**Affiliations:** Paediatrics and Clinical Epidemiology, Sitaram Bhartia Institute of Science and Research, New Delhi, India; Population Council, India Habitat Centre, New Delhi, India; Department of Epidemiology, Biostatistics and Clinical Research, Institute of Liver and Biliary Sciences, New Delhi, India; Department of Physiology, St John’s Medical College, Bengaluru, India; Population Council, India Habitat Centre, New Delhi, India

## Abstract

**Background:**

WHO’s haemoglobin cutoffs to define anemia were based on five studies of predominantly White adult populations, done over 50 years ago. Therefore, a general re-examination of the existing haemoglobin cutoffs is warranted for global application, in representative healthy populations of children and adults. Such data are scarce in low-income and middle-income countries; however, a 2019, large-scale, nationally representative survey of children and adolescents aged 0–19 years in India (Comprehensive National Nutrition Survey [CNNS]) offered an opportunity for this re-examination. Using this survey, we aimed to assess the age-specific and sex-specific percentiles of haemoglobin and cutoffs to define anaemia in the CNNS population.

**Methods:**

For this population-based study, we constructed age-specific and sex-specific haemoglobin percentiles from values reported for a defined healthy population in the CNNS, which used rigorous quality control measures during sample collection and in the laboratory analyses. To obtain a healthy population, we excluded participants with iron, folate, vitamin B12, and retinol deficiencies; inflammation; variant haemoglobins (haemoglobin A_2_ and haemoglobin S); and history of smoking. We considered age-specific and sex-specific 5th percentiles of haemoglobin derived for this healthy population as the study cutoff to define anaemia. We compared these with existing WHO cutoffs to assess significant differences between them at each year of age and sex for quantifying the prevalence of anaemia in the entire CNNS sample.

**Findings:**

Between Feb 24, 2016, and Oct 26, 2018, the CNNS survey collected blood samples from 49 486 individuals. 41 210 participants had a haemoglobin value, 8087 of whom were included in our study and comprised the primary analytical sample. Compared with existing WHO cutoffs, the study cutoffs for haemoglobin were lower at all ages, usually by 1–2 g/dL, but more so in children of both sexes aged 1–2 years and in girls aged 10 years or older. Aanemia prevalence with the study cutoffs was 19·2 percentage points lower than with WHO cutoffs in the entire CNNS sample with valid haemoglobin values across all ages and sexes (10·8% with study cutoffs *vs* 30·0% with WHO cutoffs).

**Interpretation:**

These findings support the re-examination of WHO haemoglobin cutoffs to define anaemia. Our haemoglobin reference percentiles, derived from healthy participants in a large representative Indian survey, are suitable for national use in India. Substantial variations in the 5th percentile of haemoglobin values across the 1–19 years age range and between sexes argue against constructing common cutoffs in stratified age groups for convenience.

**Funding:**

None.

## Introduction

An insufficiency in red blood cell mass to adequately deliver oxygen to peripheral tissues is defined as anaemia, from a functional perspective.^1^ An accurate case definition is crucial for both individualised clinical care and public health management.^2^ Anaemia is conventionally identified when the haemoglobin concentration in blood falls under an age-specific and sex-specific threshold or cutoff.^1,2^ However, the cutoffs proposed by WHO vary substantially from those proposed by other organisations such as the US Centres for Disease Control and Prevention (CDC), expert clinical organisations, haematology textbooks, or even individual clinical and research laboratories.^2,3^ However, the WHO cutoffs are used globally by most countries, especially to determine the burden of anaemia in a population.

The existing WHO cutoffs to define anaemia were first proposed in 1968, from five studies of predominantly white adult populations in North America and Europe;^2,4,5^ at the time, data from various ages—especially for infants, young children, adolescents, and older people—were not available. In 2000, in addition to adjustments for altitude and smoking, the age group 5–14 years was split to define a lower haemoglobin cutoff for children aged 5—11 years, on the basis of the US National Health and Nutrition Examination Survey 2 data.^6,7^ No separate cutoffs were defined for infants younger than 6 months; the reference value for age 2—6 months was added in a later WHO document.^3,8^

A 2019 review of 60 global studies, reported between 1975 and 2018, evaluated haemoglobin variation across the lifecycle.^3^ Haemoglobin cutoffs for anaemia similar to WHO recommendations were reported in several studies but tended to be lower for infants, young children, premenopausal women, and older people and higher for men. The haemoglobin cutoffs in children and adolescents from African and Asian regions were substantially lower (1–2 g/dL) in some datasets. However, limited conclusions can be made regarding the normal reference ranges because only a few studies had specifically excluded individuals with iron deficiency or inflammation.^3^ There were also uncertainties regarding the optimal testing methods to measure haemoglobin concentration.^4^ Although anaemia is defined functionally, its diagnosis is presently based on a haemoglobin cutoff that is statistically derived rather than on functional or health outcomes, which would be ideal. The present haemoglobin cutoffs for anaemia, derived 50 years ago, need reassessment to potentially enhance the evaluation of determinants, burden quantification, and the management of anaemia at the individual and population level.

WHO is indeed re-examining the appropriateness of the existing haemoglobin cutoffs to define anaemia, on the basis of relevant global evidence.^2,4^ One suggested approach is to define anaemia by haemoglobin concentrations lower than the reference range (extreme lower statistical centile, eg, the 2·5th centile) in a representative and healthy population, using stringent exclusion criteria.^4^ Such data are scarce, especially for children residing in low-income and middle-income countries (LMICs). A 2019 conference abstract reported summary haemoglobin data from 25 countries for preschool children (aged 6—59 months, 24 surveys, n=35 088) and women of reproductive age from the Biomarkers Reflecting Inflammation and Nutritional Determinants of Anaemia (BRINDA) project.^9^ Their definition of healthy was based on the exclusion of iron and vitamin A deficiency, inflammation detected through biomarkers, and malaria. In this study, we aimed to assess the age-specific and sex-specific percentiles of haemoglobin and cutoffs to diagnose anaemia in children and adolescents aged 1–19 years, including additional rigorous criteria to define a healthy population, using the 2019 quality controlled, nationally representative Comprehensive National Nutrition Survey (CNNS) in India.^10^

## Methods

### Study design and participants

The CNNS was done under the aegis of the Indian Ministry of Health and Family Welfare in collaboration with UNICEF and the Population Council (New Delhi, India). The CNNS was designed to provide nationally representative and comprehensive nutritional profiling of preschoolers (aged 0–4 years), school-age children (5–9 years), and adolescents (10–19 years), based on biological sample assessment and multiple anthropometric measures. Because blood samples were not collected for children younger than 12 months, haemoglobin percentiles were analysed in children and adolescents aged 1–19 years.

The detailed survey design and methods are published elsewhere.^10^ Briefly, the CNNS used a multistage, stratified, probability proportion to size cluster sampling design to select a nationally representative sample of households and individuals aged 0—19 years across all 29 states of India and the capital Delhi. Households with one or more individuals aged 0—19 years were randomly selected from rural and urban primary sampling units. Children or adolescent members were classified into three age strata (0—4 years, 5—9 years, and 10—19 years), and only one child or adolescent was selected from each stratum per household. Children or adolescents who had a chronic illness, physical disability, mental illness, cognitive disability, or any ongoing acute illness (eg, fever or infection) were excluded from the survey.

Ethical approvals were obtained from the Ethics Committee of the Postgraduate Institute for Medical Education and Research (Chandigarh, India) and the Institutional Review Board of the Population Council. Written, informed consent was obtained from caregivers of children aged 0—10 years. For adolescents aged 11—17 years, written informed consent was obtained from their caregivers and written informed assent obtained from the participants. Adolescents aged 18—19 years provided their own consent.

**Figure 1:**
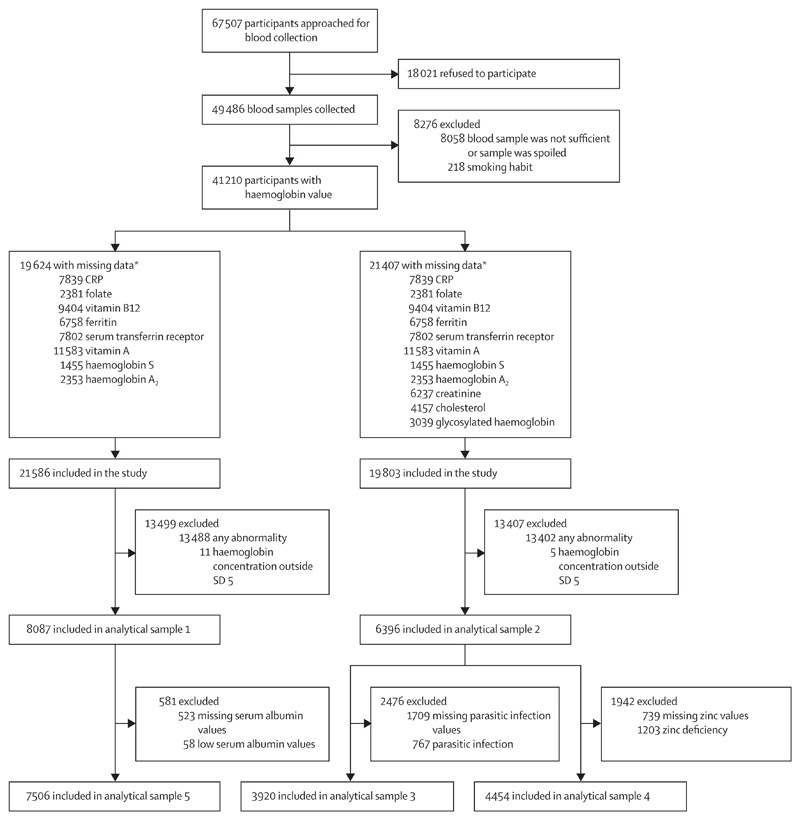
Flowchart of sequential participant exclusion for the primary and the four sensitivity analytical samples Serum albumin, creatinine, cholesterol, and glycosylated haemoglobin assessments were done only for children older than 5 years. CRP=C-reactive protein. *Not mutually exclusive.

For this population-based study, we selected one primary sample and four additional analytical samples for sensitivity analysis, applying different exclusion criteria. This strategy was adopted to optimise the sample size for improved precision without compromising on important biomarkers; the simultaneous use of all biomarkers for defining healthy participants reduced the sample size substantially. Additionally, some investigations (serum albumin, creatinine, cholesterol, and glycosylated haemoglobin concentrations) were done only for children older than 5 years. All participants aged 1—19 years with available haemoglobin values and with no history of smoking were included in the initial filter (figure 1). Among these, participants with available data for all biomarkers, namely serum CRP, ferritin, transferrin receptor, cyanocobalamin, retinol, erythrocyte folate, and variant haemoglobin (haemoglobin A_2_ and haemoglobin S) were considered, and those with abnormal values for these biomarkers (as defined in table 1) and extreme haemoglobin values (outside SD 5) were excluded, to provide the primary sample (analytical sample 1). For analytical sample 2, additional exclusion criteria included abnormally high serum creatinine, total cholesterol, and glycosylated haemoglobin. Analytical sample 3 additionally excluded participants with stool parasitic infestations and analytical sample 4 excluded those with zinc deficiency. Analytical sample 5 excludedparticipants in the primary sample who had hypo-albuminaemia. To assess if the exclusion criteria considered in analytical samples 2–5 resulted in different haemoglobin cutoffs from those of analytical sample 1, the 95% CI of the age-specific and sex-specific smoothed fifth percentiles of haemoglobin concentration from each analytical sample were examined in a sensitivity analysis to evaluate if they overlapped. Because these cutoffs were not significantly different (overlapping 95% CIs), the smoothed age-specific and sex-specific haemoglobin percentiles obtained from analytical sample 1 were used and the fifth percentile comprised the study cutoff for defining anaemia.

### Procedures used in the CNNS

Sociodemographic details and history of previous illness (including malaria) in the preceding 2 weeks were recorded in a pretested pro forma. The blood sample collection procedure and protocols followed are detailed elsewhere.^10,19^ Trained phlebotomists collected venous blood from children aged 1–4 years (8 mL) and 5–19 years (10 mL) for estimating micronutrient concentrations and biomarkers for non-communicable diseases (5–19 years only). Haemoglobin was estimated by the conventional best practice of drawing venous blood and using the cyanmethaemoglobin method of automated photometric estimation.^20^ Biochemical analyses were done in a commercial laboratory with stringent quality control measures (SRL Labs, Mumbai, Gurugram, and Kolkata, India). Rigorous control and monitoring systems were included in the standard operating procedures for quality assurance of biomarker data. First, an internal quality control sample was used for each batch of 20 survey samples. Second, for external quality assurance, a subset of samples was sent to other participating laboratories monthly for comparison testing. The SRL laboratories also participated in the Bio-Rad and CDC external quality assurance scheme. Third, on a weekly basis, a percentage of samples were split and reanalysed.^10^ Apart from haemoglobin, the following blood biomarkers, with a defined role in haematopoiesis, were used for selecting healthy participants for the purpose of defining haemoglobin cutoffs for the diagnosis of anaemia: erythrocyte folate, serum concentrations of ferritin, transferrin receptor, cyanocobalamin, retinol, zinc, C-reactive protein (CRP), creatinine, total cholesterol, albumin and glycosylated haemoglobin, and haemoglobin variants (haemoglobin S and haemoglobin A_2_). Stool samples were examined for the presence of parasites. The laboratory estimation methods for the biomarkers and the cutoffs used for specific diagnoses are summarised in table 1.^5,7,10,11–19^

### Statistical analysis

Differences in sociodemographic parameters between the entire sample of participants with a valid haemoglobin and the healthy participants chosen in analytical sample 1 were assessed with ANOVA and χ^2^ tests. We applied a generalised additive model for location scale and shape to estimate LMS (ie, skewness of distribution, median of distribution, and coefficient of variation of distribution) values and penalised smooth percentiles for altitude adjusted haemoglobin over age separately for boys and girls in analytical sample 1. We adopted the Box-Cox-Cole-Green transformation to estimate location, scale, and shape parameters.^21^ The 5th percentile of haemoglobin distribution was considered as the haemoglobin cutoff for the diagnosis of anaemia in this study. These cutoffs were compared with the existing WHO haemoglobin cutoff values to assess significant differences by evaluating if the 95% CI of the smoothed 5th percentile (haemoglobin cutoff of this study), at each age and sex, included the WHO cutoff value. Furthermore, we estimated age-specific and sex-specific anaemia prevalence using appropriate survey weights (with 95% CIs) from the entire CNNS data with valid haemoglobin samples, using cutoff values from both WHO and this study. The anaemia prevalence estimates using WHO and this study’s haemoglobin cutoffs were considered to be significantly different if their 95% CIs did not overlap. We used the statistical software R, version 3.6.1, with gamlss library for the entire analysis.

### Role of the funding source

The funder had no role in study design, data collection, data analysis, data interpretation, writing of the report, or the decision to submit for publication.

## Results

Between Feb 24, 2016, and Oct 26, 2018, the CNNS survey collected blood samples from 49 486 individuals, 41 210 of whom had a haemoglobin value. After general and sample-specific exclusions (figure 1), 8087 participants were included in the primary sample (analytical sample 1), 6396 participants in analytical sample 2, 3920 participants in analytical sample 3 (from those in sample 2), 4454 participants in analytical sample 4 (from those in sample 2), and 7506 participants in analytical sample 5 (from those in the primary sample; figure 1). Of the 8087 participants in the primary sample, only 21 (0·26%) reported a history of malaria in the 2 weeks preceding the survey.

The sociodemographic characteristics of the primary analytical sample and their comparison with those of the non-analytic sample, in three broad age groups (1–4, 5–9, and 10–19 years) are summarised in appendix 5 (pp 1–2). The exclusion criteria resulted in 80% of data loss from the initial filter. The primary analytical sample included participants of predominantly Hindu (approximately 70%), Muslim (approximately 15%), and Christian (approximately 10%) religions. The eastern, northeastern, and northern regions had 19–28% participants each, the southern region had 11–16%, and the central and western regions had 5–6% participants each. Children in the primary sample were undersized Study cutoffs are the 5th percentile from analytical sample 1 (95% CI represented by the shaded area). compared with WHO standards,^22^ with means ranging from −0·8 Z score to −1·2 Z score for various anthropometric indices. Two thirds of participants were from the two richest wealth quintiles, and the primary sample had more boys than girls (3–10% higher) and more rural than urban residents (9–10% higher). Compared with excluded participants, the analysed children were slightly older (by 0·2 years) in the 1–4 years age group and slightly younger (by 0·4 years) in the 10–19-years age group; their haemoglobin (0·2–0·5 g/dL), anthropometry (0·1–0·2 Z score), and wealth index were also higher. There were marginally more Muslim participants in the included participants than in those excluded, and minor regional variations.

The mean haemoglobin concentrations at various ages were almost identical in girls for the five analytical samples (appendix 5 p 4). Likewise, the mean concentrations were similar in boys except at ages 11–12 years, for which the value was lower in analytical sample 3 and higher in sample 4 (both by approximately 0·5 g/dL) compared with those in analytical samples 1 and 2 (overlapping 95% CIs, p>0·05 for all five samples). We plotted the smoothed 5th percentile values from each analytical sample, along with their 95% CIs across all ages (appendix 5 p 5). As stated previously, these CIs overlapped in all instances, and the primary analytical sample was used to derive anaemia cutoffs from the CNNS data and for the comparison with WHO cutoffs.

**Figure 2:**
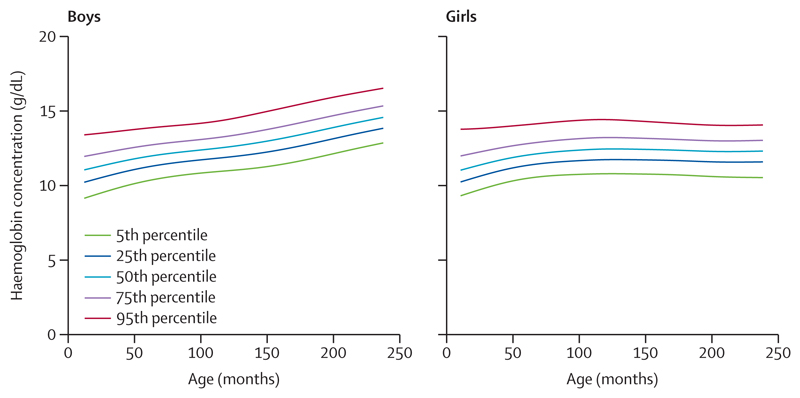
Smoothed haemoglobin percentile curves for ages 1–19 years drawn from the primary analytical sample 1

We assessed the sex-specific smoothed haemoglobin (g/dL) percentiles from ages 1–19 years in the analytical sample 1 (figure 2; appendix 5 p 3). The 50th percentile for haemoglobin rose by 1·5 g/dL between ages 1–9 years, with similar values for boys and girls. Subsequently, up to age 19 years, boys had nearly 2 g/dL higher haemoglobin concentrations, whereas these remained constant in girls. The sex difference in haemoglobin concentrations increased to 1 g/dL at age 14–15 years and widened to 2 g/dL at age 19 years. The 5th percentile cutoffs also rose by 1·5 g/dL from ages 1–8 years, with similar values across sexes. This cutoff gradually increased by 2 g/dL in boys, but remained unchanged in girls up to age 19 years.

**Figure 3:**
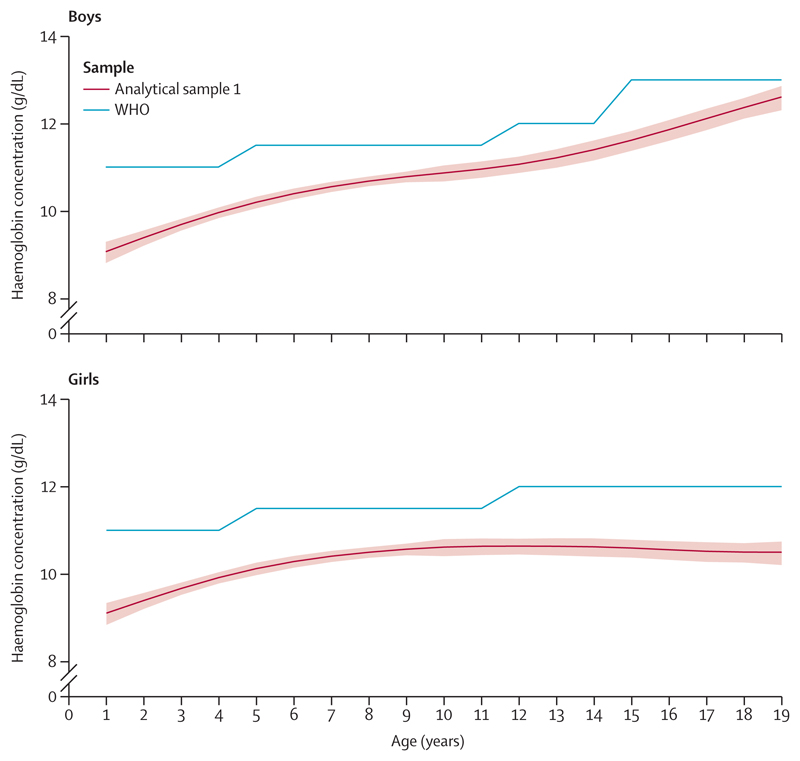
Age-specific and sex-specific study cutoffs and WHO anaemia cutoffs in children and adolescents aged 1–19 years Study cutoffs are the 5th percentile from analytical sample 1 (95% CI represented by the shaded area).

The age-specific and sex-specific haemoglobin cutoffs to define anaemia, from this secondary analysis of the CNNS, were lower throughout ages 1–19 years than the WHO recommended cutoffs. For both sexes and across the ages, the WHO cutoff was outside the 95% CI of this study’s cutoffs (figure 3). The differences between the study and WHO cutoffs narrowed from approximately 2 g/dL at age 1 year to approximately 1 g/dL at age 6 years, with subsequent variations of 0·4–1·3 g/dL up to age 19 years. By contrast, in girls, the differences between the study and WHO cutoffs narrowed from approximately 1·7 g/dL at age 1 year to approximately 1 g/dL at age 5–6 years, with subsequent variations of 0·8–1·5 g/dL up to age 19 years.

We compared the anaemia prevalence using the haemoglobin cutoffs from this study and WHO applied to all children and adolescents aged 1–19 years in the CNNS with valid haemoglobin estimates, excluding those with history of smoking (218 participants; table 2). The overall weighted anaemia prevalence was 19·2 percentage points lower with this study’s cutoffs than with WHO cutoffs (table 2). Likewise, weighted anaemia prevalence was lower with this study’s cutoffs than with WHO cutoffs for all age groups: 25·1 percentage points in ages 1—4 years, 15·3 percentage points in ages 5—9 years, 15·6 percentage points in ages 10—14 years, and 22·3 percentage points in ages 15—19 years. The differences were marked for ages 1—4 years and 15—19 years. An inspection of the 95% CIs of the prevalence estimates by both cutoffs showed that they did not overlap for any age group across both the sexes and for the overall prevalence. The difference in anaemia prevalence (the gap) between the cutoffs of this study and WHO was higher for boys aged 1—4 years (28·3 in boys *vs* 22·2 in girls), almost similar in both sexes for 5—9 years (14·5 in boys *vs* 16·1 in girls), and substantially higher for girls older than 10 years (22·6 in girls *vs* 8·9 in boys for ages 10—14 years and 32·6 in girls *vs* 11·5 in boys for ages 15—19 years).

## Discussion

Using stringent inclusion criteria to define a healthy sample, our study provides age-specific and sexspecific haemoglobin reference centiles in children and adolescents aged 1—19 years from a recent national survey in India. We used the 5th percentile value to define cutoffs to diagnose anaemia across age and sex, and these were lower than the existing WHO cutoffs for the same ages, usually by 1—2 g/dL, and more so in children aged 1—2 years and in girls aged 10 years or older. When applied to the overall sample in the CNNS with valid haemoglobin values, our study’s haemoglobin cutoffs defined a lower prevalence of anaemia in children and adolescents throughout the age range of 1—19 years compared with the anaemia prevalence derived from WHO cutoffs. The gap between the two prevalence estimates was 19·2 percentage points, with marked gaps for ages 1—4 years and 15—19 years, but a lower gap for 5—14 years. The gap in anaemia prevalence was higher for boys than girls aged 1—4 years, almost similar in both sexes for 5—9 years, and substantially higher for girls aged 10 years or older.

The finding of substantially lower percentiles and anaemia diagnostic cutoffs in our study than those recommended by WHO when using a defined healthy sample population from the CNNS is intriguing and offers an accurate and current estimate of specific haemoglobin norms for India. Haemoglobin wasestimated from venous blood by the most accurate cyan-methaemoglobin estimation method. Although mixed conclusions have emerged in various studies, datasets—including those from India in field settings—indicate a higher haemoglobin estimate (up to 0·6-0·9 g/dL) in venous blood than in capillary samples.^23,24^ Rigorous quality control procedures were adhered to in the CNNS and validation of storage and transport procedures were done separately.^10,25^ The data are fairly representative of a national sample, including rural and urban participants across all wealth quintiles. The statistical differences in sociodemographic characteristics from those excluded from our study were in the expected direction and of little practical relevance (minor effect sizes) to substantially bias the national representativeness of our primary analytical sample. The findings remained stable in various sensitivity analyses. We excluded the most common pathological conditions that contribute to lower haemoglobin concentrations in public health settings. These included inflammation, common haemo-globinopathies, haematopoietic nutrient deficiencies (hypoalbuminaemia and deficiencies in iron, folate, vitamin B12, vitamin A, and zinc), intestinal parasitosis, high risk of renal dysfunction, and cardiometabolic risk biomarkers (hypercholesterolaemia and impaired glucose homoeostasis as surrogates for risk of adiposity and the attendant inflammatory response). Subclinical malaria, though not formally examined by a peripheral smear examination, was effectively excluded through negative inflammatory biomarkers, and negligible (0·26%) history of malaria in the 2 weeks preceding the survey. We chose the 5th percentile cutoffs to define anaemia for comparability with published literature;^9^ these cutoffs are marginally higher than the suggested 2·5th percentile.^4^ The 5th percentile is also a more conservative cutoff; if the 2·5th percentile was chosen as the cutoff, the anaemia prevalence would also be lower than our estimates and further from prevalence estimates based on the WHO cutoff.

The observed age and sex patterns of haemoglobin concentrations are in consonance with global literature.^1,3^ Higher haemoglobin concentrations in boys during adolescence have been ascribed to greater muscle mass and testosterone.^1,3,26^ This aspect, as well as the onset of menstruation in girls, could partly explain the greater difference in anaemia prevalence between adolescent boys and girls. In secondary analyses from the US National Health and Nutrition Examination Surveys, the mean adult haemoglobin concentrations of Black, Hispanic, and Asian individuals were lower than those of White individuals.^27,28^ Evidence from high-income countries also suggests that haemoglobin cutoffs to define anaemia in children aged 6 months to 2 years should be lowered.^3^ Anaemia cutoffs in children and adolescents from African and Asian regions were substantially lower (1-2 g/dL) in several datasets compared with WHO recommended cutoffs; however, limited conclusions are possible on norms because few studies had specifically excluded individuals with iron deficiency or inflammation.^3^ Therefore, scarce data exist to directly compare with our findings, especially from LMICs. A conference abstract has reported summary data on preschool children (aged 6–59 months, 24 surveys, n=35 088) among selected healthy participants who had no iron or vitamin A deficiency, inflammation (CRP or α1-acid glycoprotein biomarkers), or malaria (if measured).^9^ This resulted in 17–88% data loss, and the age-adjusted and country-adjusted mean of haemoglobin concentration was 11·7 g/dL (SE 0·14), with substantial country heterogeneity (p<0·001). The haemoglobin concentration of the pooled countries at the 5th percentile was 9·4 g/dL (range 7·9 g/dL for Pakistan to 11·3 g/dL for USA). The abstract concluded that a single haemoglobin cutoff to define anaemia might not work for every country. By comparison, our study’s mean and 5th percentile of haemoglobin concentrations for children aged 1–5 years were largely comparable. The differences in exclusion criteria and age group (absence of children aged 6–12 months in CNNS) might have slightly increased our estimates.

Other plausible explanations within the physiological realm could partly account for our study’s haemoglobin cutoffs being lower than the WHO cutoffs based on White participants. Geographical differences could be partly related to the heritability of haemoglobin, which has been shown to explain 34–43% of variation in one Italian study.^29^ Children and adults living in India have been characterised by a muscle-thin but high-adipose body composition compared with those in other settings.^30–32^ Haemoglobin concentrations are positively associated with skeletal muscle density, mass, and strength, and inversely with fat mass.^26,33,34^ Estimates indicate that only 17% of children and 38% of adolescents in India achieve the recommended levels of physical activity^35,36^ and, in one subnational study, fewer than 10% of adults (aged ≥20 years) engaged in recreational physical activity.^37^ This is relevant, because the aerobic requirements for increased muscular energy expenditure provide relevant haematopoietic feedback to the bone marrow, even among athletes with good muscle mass,^38^ and the promotion of physical fitness and exercise per se elevates haemoglobin concentration.^39^ In public health settings, these unconventional factors are ignored for establishing causes and instituting interventions for anaemia. This might partly explain the observed proportion (30–48%) of “anaemia of other causes”^19^ and the relatively static burden of mild anaemia in India, despite decades of anaemia control programmes.

The following limitations of our study merit consideration. First, our stringent exclusion criteria resulted in 80% loss of sample; however, this loss is within the range observed in the BRINDA analysis we previously described.^9^ Second, we could not evaluate a few other haematopoietic nutrient deficiencies (thiamine, pyridoxine, and copper), but these are rare and unlikely to affect the estimates in public health settings. We also could not evaluate chronic inflammation through serum α1-acid glycoprotein measurements, nor could iron stores be evaluated in detail because CNNS did not collect data on these parameters. Third, the survey study design precluded the inclusion of infants (aged 0–12 months) and also the correlation with robust functional markers for validating anaemia cutoffs. Fourth, even though exposure to infections in the preceding 2 weeks was an exclusion criteria, lingering post-infectious altered erythropoiesis could not be ruled out.

India should adopt these lower haemoglobin cutoffs instead of the present WHO cutoffs for diagnosing anaemia, because of the contemporary context specificity, national representativeness, accounting for an overwhelming majority of pathological conditions, and rigorous quality control for haemoglobin estimation. These cutoffs might provide a closer estimate of the true burden of anaemia in the country than that calculated by using WHO cutoffs, its grading as a public health problem (mild instead of severe),^7^ and its responsiveness to appropriate public health interventions. A substantially lower burden than that currently estimated might also economically permit an in-depth evaluation for a precise aetiological diagnosis. Substantial variations in anaemia cutoffs with age and sex argue for use of haemoglobin centile curves, analogous to growth curves, to prevent misclassification errors. Mounting evidence exists of high heterogeneity in haemoglobin cutoffs among healthy children from different countries.^3,9^ Therefore, WHO should urgently initiate a relook at the one-size-fits-all definition of anaemia and issue updated evidencebased guidelines.

In conclusion, on the basis of high-quality national data from a recent survey, this study provides contemporary age-specific and sex-specific haemoglobin reference centiles for children and adolescents aged 1–19 years, which are suitable for national use in India. Compared with WHO recommendations, these cutoffs resulted in a substantially lower prevalence of anaemia, which is probably a more accurate reflection of the burden responsive to relevant public health interventions. These findings will contribute to WHO’s efforts to reexamine the applicability of a one-size-fits-all definition of anaemia for different regions and ethnic populations.

## Supplementary Material

Appendix 1

Appendix 2

Appendix 3

Appendix 4

Appendix 5

## Figures and Tables

**Table 1:** Biomarker estimation methods and cutoffs used for specific diagnoses

	Biomarkers (estimation method)	Cutoffs used
Anaemia (WHO, 2011)^7^	Blood haemoglobin* (cyanmethaemoglobin method, photometric estimation; LH750, Beckman Coulter, Brea, CA, USA)	WHO-based cutoffs: <11 g/dL for 1–4 years; <11·5 g/dL for 5–11 years; <12 g/dL for 12–14 years; <13 g/dL for 15–19 years, male individuals; <12 g/dL for 15–19 years, female individuals
Iron deficiency (WHO, 2020)^11^	Serum ferritin (two-site immunoassay with direct chemiluminescence; Centaur, Siemens, Chicago, IL, USA)	<12 μg/L for 1–4 years, <15 μg/L for 5–19 years
Iron deficiency (Siemens N latex serum transferrin kit-based cutoff)	Serum transferrin receptor (particle enhanced immunonephelometry; BN II, Siemens)	≥1·76 mg/L
Folate deficiency (de Benoist, 2008)^12^	Erythrocyte folate (competitive immunoassay with direct chemiluminescence; Centaur)	<151 ng/mL
Vitamin B12 deficiency (de Benoist, 2008)^12^	Serum cyanocobalamin (immunoassay with direct chemiluminescence; Advia Centaur, Siemens)	<203 pg/mL
Vitamin A deficiency (WHO, 2011b)^5^	Serum retinol (HPLC, reverse phase chromatography)	<20 μg/dL
Zinc deficiency (IZincG 2004)^13^	Serum zinc (flame atomic absorption spectrometry with deuterium correction)	<65 μg/dL for 1–9 years; for 10–19 years: <74 μg/dL for fasting male individuals, <70 μg/dL for fasting female and non-fasting male individuals, <66 μg/dL for non-fasting female individuals
High risk for renal dysfunction (Williamson, 2011)^14^	Serum creatinine (spectrophotometry, alkaline picrate—kinetic IFCC IDMS standardised)	>0·7 mg/dL for 5–12 years, >1·0 mg/dL for 13–19 years
Hypercholesterolaemia (Expert Panel, 2011)^15^	Serum total cholesterol (spectrophotometry, cholesterol oxidase esterase peroxidase)	≥200 mg/dL for 5–19 years
Impaired glucose homoeostasis including diabetes (Expert Panel, 2011)^15^	Glycosylated haemoglobin (HPLC)	>5·6% for 5–19 years
Hypoalbuminaemia (Kim et al, 2017)^16^	Serum albumin (spectrophotometry, BCP dye binding)	<3·5 g/dL for 5–19 year
Inflammation (Namaste et al, 2017)^17^	Serum CRP (particle-enhanced Immunonephelometry; BN II)	>5 mg/L
Haemoglobinopathy (Bain and Lewis, 2012)^18^	Variant haemoglobins (HPLC; CDM system, Bio-Rad, Hercules, CA, USA)	Haemoglobin A2 3·5–9·0% for the thalassaemia trait, any haemoglobin S for sickle cell
Intestinal parasitic infestation	Stool sample (concentration method and trichrome staining)	Any protozoal or helmintic ova or cyst

BCP=bromocresol purple. CRP=C-reactive protein. HPLC=high-performance liquid chromatography. IDMS=isotope dilution mass spectrometry. IFCC=International Federation of Clinical Chemistry and Laboratory Medicine.

* Haemoglobin concentrations were adjusted for altitude in survey enumeration areas higher than 1000 m.

**Table 2:** Age-specific and sex-specific anaemia prevalence using study and WHO cutoffs

	Boys			Girls			Total		
	Participants	CNNS	WHO	Participants	CNNS	WHO	Participants	CNNS	WHO
**Age (years)**									
1	1014	20·9% (16·2–26·6)	68·0% (62·7–72·9)	913	19·0% (14·9–23·9)	56·5% (49·9–62·8)	1927	20·2% (16·8–23·7)	62·5% (58·1–66·7)
2	1440	16·9% (12·7–22·0)	48·9% (43·4–54·3)	1246	25·2% (19·1–32·5)	53·3% (46·9–59·7)	2686	20·8% (16·9–25·2)	51·0% (46·7–55·3)
3	1811	9·4% (6·9–12·6)	36·9% (31·8–42·2)	1549	17·5% (13·9–21·8)	38·6% (33·3–44·3)	3360	13·1% (10·6–15·9)	37·7% (33·8–41·7)
4	1940	9·1% (6·8–11·9)	24·3% (20·2–28·9)	1707	14·0% (10·9–17·8)	26·6% (21·8–32·0)	3647	11·6% (9·5–14·1)	25·5% (22·1–29·1)
5	1427	5·6% (3·5–8·8)	31·5% (26·8–36·6)	1365	10·4% (8·1–13·1)	32·7% (28·2–37·5)	2792	8·0% (6·3–9·9)	32·1% (28·6–35·7)
6	1596	9·5% (5·8–15·2)	26·1% (21·5–31·4)	1452	7·5% (5·5–10·2)	28·0% (23·3–33·1)	3048	8·6% (6·3–11·5)	27·0% (23·2–31·2)
7	1646	9·8% (7·3–12·9)	22·8% (19·1–26·9)	1465	8·1% (5·2–12·5)	26·7% (20·6–33·8)	3111	8·9% (6·6–11·8)	24·8% (20·9–29·1)
8	1788	5·7% (4·1–7·8)	16·3% (13·3–19·9)	1535	9·4% (6·7–12·9)	19·4% (15·5–23·9)	3323	7·5% (5·9–9·5)	17·8% (15·3–20·6)
9	1562	7·9% (5·9–10·5)	15·3% (12·3–18·9)	1302	7·7% (5·4–10·9)	17·2% (13·8–21·2)	2864	7·8% (6·2–9·8)	16·2% (13·8–18·9)
10	786	9·4% (5·8–14·8)	17·0% (12·5–22·7)	682	5·4% (3·5–8·2)	19·4% (14·2–25·9)	1468	7·5% (5·3–10·6)	18·1% (14·6–22·2)
11	798	8·1% (5·4–11·9)	12·3% (8·9–16·7)	766	7·8% (5·3–11·6)	19·5% (14·7–25·4)	1564	8·0% (5·9–10·6)	15·8% (12·6–19·5)
12	863	5·6% (3·5–8·7)	18·1% (13·7–23·7)	795	10·0% (6·9–14·2)	39·9% (31·2–49·2)	1658	7·7% (5·7–10·3)	28·7% (24·4–33·4)
13	806	7·5% (4·3–12·4)	21·2% (16·1–27·5)	739	13·0% (9·4–17·6)	38·4% (32·2–45·1)	1545	10·0% (7·5–13·2)	29·1% (24·9–33·6)
14	801	11·4% (6·7–18·8)	16·0% (10·7–23·1)	742	11·5% (6·7–19·2)	39·9% (32·4–48·1)	1543	11·5% (7·8–16·5)	29·0% (24·1–34·4)
15	801	7·3% (5·1–10·3)	31·2% (25·6–37·4)	785	11·1% (8·3–14·6)	47·4% (40·3–54·5)	1586	9·3% (7·5–11·5)	39·8% (35·2–44·6)
16	725	8·2% (5·2–12·7)	26·5% (20·0–34·1)	747	13·2% (9·7–17·8)	48·5% (40·7–56·3)	1472	10·9% (8·4–13·9)	38·0% (32·9–43·3)
17	637	5·6% (3·2–9·6)	10·9% (7·4–15·8)	689	21·5% (14·8–30·2)	49·1% (41·1–57·2)	1326	14·2% (10·2–19·5)	31·6% (26·6–37·0)
18	639	6·5% (3·8–10·7)	8·8% (5·7–13·2)	625	14·2% (10·0–19·8)	46·9% (38·6–55·3)	1264	10·2% (7·7–13·5)	27·4% (22·4–33·0)
19	523	6·4% (4·1–9·8)	11·1% (7·1–17·1)	503	15·8% (11·2–21·9)	44·8% (37·2–52·7)	1026	10·7% (8·1–14·0)	26·6% (22·1–31·6)
**Age groups (years)**									
1–4	6205	12·3% (10·9–14·9)	40·6% (37·9–43·2)	5415	18·3% (16·1–20·6)	40·5% (37·8–43·3)	11 620	15·4% (14·0–16·9)	40·5% (38·5–42·6)
5–9	8019	7·7% (6·4–9·3)	22·2% (20·2–24·3)	7119	8·6% (7·4–9·9)	24·7% (22·4–27·2)	15 138	8·2% (7·1–9·3)	23·5% (21·8–25·2)
10–14	4054	8·2% (6·6–10·1)	17·1% (14·8–19·6)	3724	9·7% (7·9–11·8)	32·3% (28·8–35·9)	7778	8·9% (7·7–10·3)	24·5% (22·5–26·6)
15–19	3325	6·8% (5·6–8·4)	18·3% (15·9–21·0)	3349	14·9% (12·7–17·5)	47·5% (43·9–51·1)	6674	11·0% (9·6–12·5)	33·3% (30·9–35·7)
**Overall**									
1–19 years	21 603	9·1% (8·3–10·1)	25·9% (24·5–27·3)	19 607	12·6% (11·6–13·5)	34·4% (32·8–36·1)	41 210	10·8% (10·1–11·5)	30·0% (28·9–31·3)

Data are n or percentage (95% CI). Weighted anaemia prevalence estimates from the CNNS sample, excluding participants with a smoking habit (n=218). CNNS=Comprehensive National Nutrition Survey.

## Data Availability

The Indian Ministry of Health and Family Welfare owns the CNNS data. Data used in this paper were released for public use by the Ministry and UNICEF. The dataset is available on request from Dr Rajib Acharya (racharya@popcouncil.org).

## Research in context

### Evidence before this study

In 2017, WHO re-examined the appropriateness of existing haemoglobin thresholds to define anaemia. One suggested approach was to define anaemia by haemoglobin concentrations lower than the reference range (2·5th centile) in a representative and healthy population. Such data are scarce, especially for children residing in low-income and middle-income countries. We searched PubMed and Google Scholar for literature published in English between Jan 1, 2000, and Aug 20, 2020, on haemoglobin cutoffs using the search terms “haemoglobin reference”, “haemoglobin centiles”, “haemoglobin percentiles”, “India”, and “children and adolescents”. Additionally, we searched WHO technical reports for the diagnosis of anaemia. In a review of 60 studies done between 1975 and 2018, Jorgensen and colleagues reported haemoglobin variation across the lifecycle and specifically lower cutoffs in studies with infants, young children, premenopausal women, and older people; however, only a few studies excluded participants with iron deficiency and inflammation. A recent conference abstract reported summary haemoglobin data from surveys in 25 countries, excluding India, for preschool children in whom iron and vitamin A deficiency, inflammation, and malaria were excluded. In view of high heterogeneity, the authors concluded that a single haemoglobin cutoff for anaemia might not work for every country. We could not locate any nationally representative studies providing agespecific and sex-specific percentiles for haemoglobin for healthy Indian children and adolescents.

### Added value of this study

We report for the first time age-specific and sex-specific haemoglobin percentiles from a nationally representative sample of children and adolescents aged 1–19 years in India, using a stringent definition of a healthy population that excludes iron, folate, vitamin B12, and retinol deficiencies; inflammation (C-reactive protein); variant haemoglobins (haemoglobin A_2_ and haemoglobin S); and history of smoking. Sensitivity analyses after further excluding participants with high total cholesterol, high glycosylated haemoglobin, high serum creatinine, zinc deficiency, stool parasitic infestation, and hypoalbuminaemia yielded almost identical results. Compared with the WHO recommendation, our haemoglobin cutoffs (fifth percentile) to define anaemia were lower throughout ages 1–19 years and more so in children aged 1–2 years and in girls aged 10 years or older. The overall anaemia prevalence thus defined was lower than that estimated by the WHO cutoff, with marked differences for ages 1–4 years and 15–19 years, but a lower gap for 5–14 years.

### Implications of all the available evidence

These contemporary age-specific and sex-specific haemoglobin reference centiles for children and adolescents aged 1–19 years are suitable for national use in India. The lower prevalence of anaemia probably reflects better the burden responsive to public health interventions and could explain the relatively static prevalence of mild anaemia in India, with WHO recommended cutoffs, despite decades of preventive efforts. These findings can contribute to WHO’s efforts to re-examine the applicability of a one-size-fits-all definition of anaemia for different regions and ethnic populations.
