# Supplementary material for: Haemoglobin thresholds to define anaemia in a national sample of healthy children and adolescents aged 1–19 years in India: a population-based study
Source: Lancet Glob Health. Author manuscript; Available in PMC 2022 Jul 5. (PMC7612991; doi:10.1016/S2214-109X(21)00077-2)
Supplement: Appendix 1 [file EMS146378-supplement-Appendix_1.pdf]

THE LANCET
Global Health

Supplementary appendix 1

This translation in Hindi was submitted by the authors and we reproduce it as supplied. It has not been peer reviewed. *The Lancet*'s editorial processes have only been applied to the original in English, which should serve as reference for this manuscript.

Supplement to: Sachdev HS, Porwal A, Acharya R, et al. Haemoglobin thresholds to define anaemia in a national sample of healthy children and adolescents aged 1–19 years in India: a population-based study. *Lancet Glob Health* 2021; published online April 16. https://doi.org/10.1016/S2214-109X(21)00077-2.

हिंदी में यह अनुवाद लेखकों द्वारा प्रस्तुत किया गया था और हम इसे जैसे उपलब्ध कराया गया वैसे पुन: पेश करते हैं। इस पर सहकर्मी की समीक्षा नहीं की गई है। लैंसेट की संपादकीय प्रक्रियाओं को केवल अंग्रेजी में मूल पर लागू किया गया है, जो इस पांडुलिपि के संदर्भ के रूप में काम आना चाहिए।

भारत में 1-19 साल के बच्चों और किशोरों के राष्ट्रीय नमूने में एनीमिया को परिभाषित करने के लिए हीमोग्लोबिन थ्रेसहोल्ड: जनसंख्या आधारित अध्ययन

सारांश

पृष्ठभूमि

एनीमिया को परिभाषित करने के लिए विश्व स्वास्थ्य संगठन (डबल्यूएचओ) के हीमोग्लोबिन कट-ऑफ मुख्यतः सफेद वयस्क आबादी के पाँच अध्ययनों पर आधारित थे, जो पचास साल पहले किए गए थे। इसलिए बच्चों और वयस्कों के लिए मौजूदा हीमोग्लोबिन कट-ऑफ की पुन: परीक्षा स्वस्थ आबादी में करने की जरूरत हैं, जो वैश्विक स्तर पर लागू हो सके| ऐसे आंकड़े निम्न- और मध्य-आय-देशों में बहुत कम हैं; हालाँकि, हाल ही में, बड़े पैमाने पर, 0-19 वर्षीय भारतीय बच्चों और किशोरों के राष्ट्रीय स्तर के प्रतिनिधि सर्वेक्षण (व्यापक राष्ट्रीय पोषण सर्वेक्षण, सीएनएनएस) ने इस पुन: परीक्षा के लिए एक अवसर प्रदान किया हैं| इस सर्वेक्षण का उपयोग करते हुए, हमने आयु-विशिष्ट और यौन-विशिष्ट हीमोग्लोबिन प्रतिशत और कट-ऑफ का आकलन किया जिससे सीएनएनएस सर्वेक्षण की आबादी में एनीमिया प्रसार का पता चले।

क्रियाविधि

आयु और सेक्स-विशिष्ट हीमोग्लोबिन प्रतिशत का निर्माण सीएनएनएस में एक चयनित "स्वस्थ आबादी" के लिए रिपोर्ट किए गए मूल्यों से किया गया था, जो की संग्रह के दौरान और प्रयोगशाला विश्लेषणों के दौरान कठोर गुणवत्ता नियंत्रण उपायों की देखरेक मे किए गए थे। स्वस्थ आबादी के नमूनो के प्राप्त करने के लिए आयरन, फोलेट, विटामिन बी 12 और रेटिनोल की कमी, सूजन, वैरिएंट हीमोग्लोबिन (HbA2 और HbS), और धूम्रपान करने वालों नमूनों को छोड़ा गया था। हमने इस स्वस्थ आबादी के लिए हीमोग्लोबिन की आयु और लिंग के विशिष्ट पांचवे प्रतिशत को एनीमिया को परिभाषित करने के लिए अध्ययन कट-ऑफ के रूप में माना, और मौजूदा डबल्यूएचओ कट-ऑफ के साथ इसकी तुलना करके, पूरे सीएनएनएस नमूने में एनीमिया की व्यापकता को निर्धारित किया।

परिणाम

24 फरवरी 2016 और 26 अक्टूबर 2018 के बीच, सीएनएनएस सर्वेक्षण मे 49486 व्यक्तियों से रक्त के नमूने एकत्र किए। 41210 प्रतिभागियों में हीमोग्लोबिन का मूल्य था, जिनमें से 8087 हमारे अध्ययन में प्राथमिक विश्लेषणात्मक नमूना के रूप मे शामिल है| मौजूदा डबल्यूएचओ कट-ऑफ की तुलना में, हीमोग्लोबिन के लिए अध्ययन कट-ऑफ सभी उम्र में कम था, आमतौर पर 1-2 g/dl, लेकिन 1-2 साल के बच्चों में और 10 साल या उससे अधिक उम्र की लड़कियों में आधिक था। अध्ययन कट-ऑफ के

अनुसार एनीमिया की व्यापकता सभी उम्र और लिंगों में मान्य डबल्यूएचओ मूल्यों के साथ पूरे सीएनएनएस नमूने में 19.2% कम थी (वर्तमान अध्ययन बनाम डबल्यूएचओ कट-ऑफ में क्रमशः 10.8% बनाम 30.0%)।

व्याख्या

ये निष्कर्ष एनीमिया को परिभाषित करने के लिए डबल्यूएचओ हीमोग्लोबिन कटऑफ की पुन: परीक्षा का समर्थन करते हैं। एक बड़े प्रतिनिधि भारतीय सर्वेक्षण में स्वस्थ प्रतिभागियों से प्राप्त वर्तमान हीमोग्लोबिन संदर्भ प्रतिशत, भारत में राष्ट्रीय उपयोग के लिए उपयुक्त हैं। 1-19 वर्षों में हीमोग्लोबिन मूल्यों के पांचवे प्रतिशत में पर्याप्त विविधताएं आयु सीमा और लिंग के बीच सुविधा के लिए स्तरीकृत आयु समूहों में कटऑफ के निर्माण के खिलाफ तर्क देते हैं|

अनुदान

कोई नही|
