# Supplementary material for: Haemoglobin thresholds to define anaemia in a national sample of healthy children and adolescents aged 1–19 years in India: a population-based study
Source: Lancet Glob Health. Author manuscript; Available in PMC 2022 Jul 5. (PMC7612991; doi:10.1016/S2214-109X(21)00077-2)
Supplement: Appendix 2 [file EMS146378-supplement-Appendix_2.pdf]

THE LANCET
Global Health

Supplementary appendix 2

This translation in Punjabi was submitted by the authors and we reproduce it as supplied. It has not been peer reviewed. *The Lancet*'s editorial processes have only been applied to the original in English, which should serve as reference for this manuscript.

Supplement to: Sachdev HS, Porwal A, Acharya R, et al. Haemoglobin thresholds to define anaemia in a national sample of healthy children and adolescents aged 1–19 years in India: a population-based study. *Lancet Glob Health* 2021; published online April 16. https://doi.org/10.1016/S2214-109X(21)00077-2.

'[ਇੱਥੇ ਭਾਸ਼ਾ ਲਿਖੋ] ਵਿੱਚ ਇਹ ਅਨੁਵਾਦ ਲੇਖਕਾਂ ਦੁਆਰਾ ਪੇਸ਼ ਕੀਤਾ ਗਿਆ ਸੀ ਅਤੇ ਅਸੀਂ ਸਪਲਾਈ ਕੀਤੇ ਵਜੋਂ ਇਸਨੂੰ ਮੁੜ ਪੇਸ਼ ਕਰਦੇ ਹਾਂ। ਇਸ ਦੀ ਪੀਅਰ ਸਮੀਖਿਆ ਨਹੀਂ ਕੀਤੀ ਗਈ ਹੈ। ਲੈਂਸੈੱਟ ਦੀਆਂ ਸੰਪਾਦਕੀ ਪ੍ਰਕਿਰਿਆਵਾਂ ਮੂਲ ਤੌਰ 'ਤੇ ਕੇਵਲ ਅੰਗਰੇਜ਼ੀ ਭਾਸ਼ਾ ਵਿੱਚ ਲਾਗੂ ਕੀਤੀਆਂ ਗਈਆਂ ਹਨ, ਜੋ ਇਸ ਖਰੜੇ ਦੇ ਹਵਾਲੇ ਵਜੋਂ ਕੰਮ ਕਰੇਗੀ।'

ਭਾਰਤ ਵਿੱਚ 1-19 ਸਾਲ ਦੇ ਸਿਹਤਮੰਦ ਬੱਚਿਆਂ ਅਤੇ ਕਿਸ਼ੋਰਾਂ ਦੇ ਰਾਸ਼ਟਰੀ ਨਮੂਨੇ ਵਿੱਚ ਅਨੀਮੀਆ ਪਰਿਭਾਸ਼ਤ ਕਰਨ ਲਈ ਹੀਮੋਗਲੋਬਿਨ ਪ੍ਰਭਾਵ-ਸੀਮਾ: ਇੱਕ ਆਬਾਦੀ ਅਧਾਰਤ ਅਧਿਐਨ

ਸਾਰ

ਪਿਛੋਕੜ

ਅਨੀਮੀਆ ਪਰਿਭਾਸ਼ਤ ਕਰਨ ਲਈ ਵਿਸ਼ਵ ਸਿਹਤ ਸੰਗਠਨ (ਡਬਲਯੂਐਚਓ) ਦੇ **ਹੀ**ਮੋਗਲੋਬਿਨ ਕੱਟ-ਆਫਸ ਪੰਜਾਹ ਸਾਲ ਪਹਿਲਾਂ ਕਰਵਾਏ ਗਏ, ਮੁੱਖ ਤੌਰ ਤੇ ਚਿੱਟੇ ਬਾਲਗਾਂ ਦੀ ਆਬਾਦੀ ਦੇ ਪੰਜ ਅਧਿਐਨਾਂ 'ਤੇ ਅਧਾਰਤ ਸਨ। ਇਸ ਲਈ ਮੌਜੂਦਾ ਹੀਮੋਗਲੋਬਿਨ ਕੱਟ-ਆਫਸ ਦੀ ਵਿਸ਼ਵਵਿਆਪੀ ਵਰਤੋਂ ਲਈ, ਬੱਚਿਆਂ ਅਤੇ ਬਾਲਗਾਂ ਦੀ ਪ੍ਰਤੀਨਿਧੀ ਸਿਹਤਮੰਦ ਆਬਾਦੀਆਂ ਵਿੱਚ, ਮੁੜ ਜਾਂਚ ਦੀ ਜ਼ਰੂਰਤ ਹੈ।
ਘੱਟ ਅਤੇ ਮੱਧ–ਆਮਦਨੀ ਵਾਲੇ ਦੇਸ਼ਾਂ ਵਿੱਚ ਅਜਿਹੇ ਅੰਕੜੇ ਬਹੁਤ ਘੱਟ ਹਨ; ਪਰ, 0-19 ਸਾਲ-ਦੇ ਭਾਰਤੀ ਬੱਚਿਆਂ ਅਤੇ ਕਿਸ਼ੋਰਾਂ (ਕੌਮਪ੍ਰੀਹੈਨਸਿਵ ਨੈਸ਼ਨਲ ਨਯੂਟਰਿਸ਼ਨ ਸਰਵੇ, ਸੀਐੱਨਐੱਨਐੱਸ) ਦੇ ਇੱਕ ਤਾਜ਼ਾ, ਵੱਡੇ ਪੱਧਰ 'ਤੇ, ਰਾਸ਼ਟਰੀ ਪ੍ਰਤੀਨਿਧ ਸਰਵੇਖਣ ਨੇ ਇਸ ਦੀ ਦੁਬਾਰਾ ਪ੍ਰੀਖਿਆ ਲਈ ਇੱਕ ਮੌਕਾ ਪੇਸ਼ ਕੀਤਾ।

ਪ੍ਰਣਾਲੀ

ਸੀਐੱਨਐੱਨਐੱਸ ਵਿੱਚ ਇੱਕ ਚੁਣੀ ਗਈ "ਸਿਹਤਮੰਦ ਆਬਾਦੀ" ਲਈ ਦਰਸਾਏ ਗਏ ਮੁੱਲਾਂ ਤੋਂ ਉਮਰ ਅਤੇ ਲਿੰਗ ਨਾਲ ਵਿਸ਼ੇਸ਼ ਤੌਰ ਤੇ ਸੰਬੰਧੀ ਐਚਬੀ ਪਰਸੈਂਟਾਈਲ ਦਾ ਨਿਰਮਾਣ ਕੀਤਾ ਗਿਆ ਸੀ; ਸੀਐੱਨਐੱਨਐੱਸ ਵਿੱਚ ਨਮੂਨਾ ਇਕੱਠਾ ਕਰਨ ਦੌਰਾਨ ਅਤੇ ਪ੍ਰਯੋਗਸ਼ਾਲਾ ਦੇ ਵਿਸ਼ਲੇਸ਼ਣ ਵਿੱਚ ਸਖ਼ਤ ਗੁਣਵੱਤਾ ਨਿਯੰਤਰਣ ਉਪਾਅ ਲਗਾਏ ਸਨ। ਸਿਹਤਮੰਦ ਆਬਾਦੀ ਨੂੰ ਆਇਰਨ, ਫੋਲੇਟ, ਵਿਟਾਮਿਨ ਬੀ 12 ਅਤੇ ਰੇਟਿਨੌਲ ਦੀ ਘਾਟ, ਇਨਫਲੇਮੇਸ਼ਨ,ਵੇਰੀਐਂਟ ਹੀਮੋਗਲੋਬਿਨ (ਐਚਬੀਏ2 ਅਤੇ ਐਚਬੀਐਸ), ਅਤੇ ਤਮਾਕੂਨੋਸ਼ੀ ਕਰਨ ਵਾਲੇ ਨਮੂਨਿਆਂ ਨੂੰ ਛੱਡ ਕੇ ਪ੍ਰਾਪਤ ਕੀਤਾ ਗਿਆ ਸੀ। ਅਸੀਂ ਇਸ ਸਿਹਤਮੰਦ ਆਬਾਦੀ ਤੋਂ ਪ੍ਰਾਪਤ ਕੀਤੀ ਉਮਰ ਅਤੇ ਲਿੰਗ ਸੰਬੰਧੀ 5 ਵੀਂ ਪਰਸੈਂਟਾਈਲ ਨੂੰ ਅਨੀਮੀਆ ਪਰਿਭਾਸ਼ਤ ਕਰਨ ਲਈ ਅਧਿਐਨ ਕੱਟ-ਆਫ ਮਨਿਆ, ਅਤੇ ਪੂਰੇ ਸੀਐੱਨਐੱਨਐੱਸ ਨਮੂਨੇ ਵਿਚ ਅਨੀਮੀਆ ਦੇ ਪ੍ਰਚਲਤ ਹੋਣ ਦੀ ਮਾਤਰਾ ਨਿਰਧਾਰਤ ਕਰਨ ਲਈ ਇਹਨਾਂ ਦੀ ਮੌਜੂਦਾ ਡਬਲਯੂਐਚਓ ਕੱਟ-ਆਫਸ ਨਾਲ ਤੁਲਨਾ ਕੀਤੀ।

ਨਤੀਜੇ

24 ਫਰਵਰੀ 2016 ਤੋਂ 26 ਅਕਤੂਬਰ 2018 ਦੇ ਵਿਚਕਾਰ, ਸੀਐੱਨਐੱਨਐੱਸ ਦੇ ਸਰਵੇਖਣ ਵਿੱਚ 49,486 ਵਿਅਕਤੀਆਂ ਤੋਂ ਖ਼ੂਨ ਦੇ ਨਮੂਨੇ ਇਕੱਤਰ ਕੀਤੇ ਗਏ। 41,210 ਭਾਗੀਦਾਰਾਂ ਦਾ ਹੀਮੋਗਲੋਬਿਨ ਮੁੱਲ ਸੀ, ਜਿਨ੍ਹਾਂ ਵਿਚੋਂ 8087 ਨੂੰ ਸਾਡੇ ਅਧਿਐਨ ਵਿੱਚ ਪ੍ਰਾਇਮਰੀ ਵਿਸ਼ਲੇਸ਼ਣ ਦੇ ਨਮੂਨੇ ਵਜੋਂ ਸ਼ਾਮਲ ਕੀਤਾ ਗਿਆ। ਮੌਜੂਦਾ

ਡਬਲਯੂਐਚਓ ਦੇ ਕੱਟ-ਆਫ ਦੇ ਮੁਕਾਬਲੇ, ਹਰ ਉਮਰ ਵਿੱਚ ਹੀਮੋਗਲੋਬਿਨ ਲਈ ਅਧਿਐਨ ਕੱਟ-ਆਫ ਘੱਟ ਸਨ, ਆਮ ਤੌਰ 'ਤੇ 1-2 ਗ੍ਰਾਮ / ਡੀਐਲ ਦੁਆਰਾ, ਖਾਸ ਕਰਕੇ 1-2 ਸਾਲ ਦੀ ਉਮਰ ਦੇ ਬੱਚਿਆਂ ਅਤੇ 10 ਸਾਲ ਜਾਂ ਇਸ ਤੋਂ ਵੱਧ ਉਮਰ ਦੀਆਂ ਲੜਕੀਆਂ ਵਿਚ। ਅਧਿਐਨ ਦੇ ਕੱਟ-ਆਫਸ ਦੇ ਨਾਲ ਅਨੀਮੀਆ ਦੇ ਪ੍ਰਚਲਤ ਹੋਣ ਦੀ ਮਾਤਰਾ ਸਾਰੇ ਸੀਐੱਨਐੱਨਐੱਸ ਦੇ ਨਮੂਨੇ ਵਿਚ ਸਬ ਉਮਰਾਂ ਅਤੇ ਲਿੰਗਾਂ ਲਈ 19.2% ਘੱਟ ਸੀ (ਅਧਿਐਨ ਕੱਟ-ਆਫਸ ਵਿਚ 10.8% ਅਤੇ ਮੌਜੂਦਾ ਡਬਲਯੂਐਚਓ ਕੱਟ-ਆਫਸ ਨਾਲ 30.0%)।

ਵਿਆਖਿਆ

ਇਹ ਨਤੀਜੇ ਅਨੀਮੀਆ ਦੀ ਪਰਿਭਾਸ਼ਾ ਲਈ ਡਬਲਯੂਐਚਓ **ਹੀ**ਮੋਗਲੋਬਿਨ ਦੇ ਕੱਟ-ਆਫਸ ਦਾ ਤੁਰੰਤ ਮੁੜ ਮੁਆਇਨਾ ਕਰਨ ਦਾ ਸਮਰਥਨ ਕਰਦੇ ਹਨ। ਮੌਜੂਦਾ ਐਚਬੀ ਸੰਦਰਭ ਪਰਸੈਂਟਾਈਲ, ਜੋ ਇੱਕ ਵਿਸ਼ਾਲ ਪ੍ਰਤੀਨਿਧੀ ਭਾਰਤੀ ਸਰਵੇਖਣ ਵਿੱਚ ਸਿਹਤਮੰਦ ਭਾਗੀਦਾਰਾਂ ਤੋਂ ਲਏ ਗਏ ਹਨ, ਭਾਰਤ ਵਿੱਚ ਰਾਸ਼ਟਰੀ ਵਰਤੋਂ ਲਈ ਉਚਿਤ ਹਨ। 1-19 ਸਾਲ-ਦੇ ਨਮੂਨੇ ਵਿੱਚ, ਉਮਰ ਅਤੇ ਲਿੰਗ ਨਾਲ ਵਿਸ਼ੇਸ਼ ਤੌਰ ਤੇ ਸੰਬੰਧੀ **ਹੀ**ਮੋਗਲੋਬਿਨ ਦੀ 5 ਵੀਂ ਪਰਸੈਂਟਾਈਲ ਦੇ ਮੁੱਲਾਂ ਵਿੱਚ ਮਹੱਤਵਪੂਰਣ ਭਿੰਨਤਾਵਾਂ ਹਨ ਜੋ ਸਹੂਲਤ ਲਈ ਉਮਰ-ਸਮੂਹਾਂ ਵਿੱਚ ਆਮ ਕੱਟ-ਆਫਸ ਬਣਾਉਣ ਦੇ ਵਿਰੁੱਧ ਜਾਂਦਿਆਂ ਹਨ।

ਫੰਡਿੰਗ: ਕੋਈ ਨਹੀਂ
