# Supplementary material for: Haemoglobin thresholds to define anaemia in a national sample of healthy children and adolescents aged 1–19 years in India: a population-based study
Source: Lancet Glob Health. Author manuscript; Available in PMC 2022 Jul 5. (PMC7612991; doi:10.1016/S2214-109X(21)00077-2)
Supplement: Appendix 3 [file EMS146378-supplement-Appendix_3.pdf]

THE LANCET
Global Health

Supplementary appendix 3

This translation in Tamil was submitted by the authors and we reproduce it as supplied. It has not been peer reviewed. *The Lancet*'s editorial processes have only been applied to the original in English, which should serve as reference for this manuscript.

Supplement to: Sachdev HS, Porwal A, Acharya R, et al. Haemoglobin thresholds to define anaemia in a national sample of healthy children and adolescents aged 1–19 years in India: a population-based study. *Lancet Glob Health* 2021; published online April 16. https://doi.org/10.1016/S2214-109X(21)00077-2.

தமிழில் இந்த மொழிபெயர்ப்பு ஆசிரியர்களால் சமர்ப்பிக்கப்பட்டது, நாங்கள் அதை வழங்கியபடி மீண்டும் உருவாக்குகிறோம்.இது மதிப்பாய்வு செய்யப்படவில்லை. லான்செட்டின் தலையங்க செயல்முறைகள் ஆங்கிலத்தில் அசலுக்கு மட்டுமே பயன்படுத்தப்பட்டுள்ளன, இது இந்த கையெழுத்துப் பிரதிக்கான குறிப்பாக செயல்பட வேண்டும்.

இந்தியாவின் 1-19 வயதுடைய ஆரோக்கியமான குழந்தைகள் மற்றும் இளம் பருவத்தினரின் தேசிய மாதிரியின் மூலம் இரத்த சோகையை வரையறுக்க ஹீமோகுளோபின் குறியீடுகள் கணக்கெடுப்பு: ஒரு மக்கள் தொகை அடிப்படையிலான ஆய்வு

பின்புலம்: இரத்த சோகையை வரையறுக்க WHO இன் ஹீமோகுளோபின் அளவு 50 ஆண்டுகளுக்கு முன்பு செய்யப்பட்ட வெள்ளையர் வயது வந்தோரின் ஐந்து ஆய்வுகளின் அடிப்படையில் அமைந்தன. ஆகையால், குழந்தைகள் மற்றும் பெரியவர்களின் ஆரோக்கியமான மக்கள்தொகையில், தற்போதுள்ள ஹீமோகுளோபின் அளவுபொதுவான மறு ஆய்வு உலகளாவிய பயன்பாட்டிற்கு உத்தரவாதம் அளிக்கப்படுகிறது. இத்தகைய தகவல்கள் குறைந்த வருமானம் மற்றும் நடுத்தர வருமானம் கொண்ட நாடுகளில் குறைவு; எவ்வாறாயினும், இந்தியாவில் 0–19 வயதுடைய குழந்தைகள் மற்றும் இளம் பருவத்தினரின் 2019, பெரிய அளவிலான, தேசிய அளவில் பிரதிநிதித்துவ கணக்கெடுப்பு (விரிவான தேசிய ஊட்டச்சத்து ஆய்வு [சி.என்.என்.எஸ்]) இந்த மறு ஆய்வுக்கு ஒரு வாய்ப்பை வழங்கியது. இந்த கணக்கெடுப்பைப் பயன்படுத்தி, சி.என்.என்.எஸ் மக்கள்தொகையில் இரத்த சோகையைக் கண்டறிய ஹீமோகுளோபின் மற்றும் மதிப்பீடு (Cutoff) வயது-குறிப்பிட்ட மற்றும் பாலின-குறிப்பிட்ட சதவீதங்களை மதிப்பிடுவதை நோக்கமாகக் கொண்டோம்.

ஆராய்ச்சி முறை: இந்த மக்கள்தொகை அடிப்படையிலான ஆய்வுக்காக, சி.என்.என்.எஸ்ஸில் வரையறுக்கப்பட்ட ஆரோக்கியமான மக்கள்தொகைக்கு அறிக்கையிடப்பட்ட மதிப்புகளிலிருந்து வயது-குறிப்பிட்ட மற்றும் பாலின-குறிப்பிட்ட ஹீமோகுளோபின் சதவிகிதங்களை நாங்கள் உருவாக்கினோம், இது மாதிரி சேகரிப்பின் போது மற்றும் ஆய்வக பகுப்பாய்வுகளில் கடுமையான தரக் கட்டுப்பாட்டு நடவடிக்கைகளைப் பயன்படுத்தியது. ஆரோக்கியமான மக்கள் தொகை என்று அழைக்கப்படுவதற்கு, இரும்பு, ஃபோலேட், வைட்டமின் பி 12 மற்றும் ரெட்டினோல் குறைபாடுகளுடன் பங்கேற்பாளர்களை நாங்கள் விலக்கினோம்; வீக்கம், மாறுபாடு ஹீமோகுளோபின்கள் (ஹீமோகுளோபின் ஏ 2 மற்றும் ஹீமோகுளோபின் எஸ்), மற்றும் புகைப்பழக்கத்தின் வரலாறு. இந்த ஆரோக்கியமான மக்களுக்காக பெறப்பட்ட ஹீமோகுளோபினின் வயது-குறிப்பிட்ட மற்றும் பாலின-குறிப்பிட்ட 5 சதவிகிதத்தை இரத்த சோகையை வரையறுப்பதற்கான ஆய்வு மதிப்பீடு (cutoff)என்று நாங்கள் கருதினோம். ஒவ்வொரு வயதிலும் பாலினத்திலும் அவற்றுக்கிடையேயான குறிப்பிடத்தக்க வேறுபாடுகளை மதிப்பிடுவதற்கும் முழு சி.என்.என்.எஸ் மாதிரியில் இரத்த சோகை பரவுவதை அளவிடுவதற்கும் தற்போதுள்ள WHO மதிப்பீடு உடன்(cutoff) ஒப்பிட்டோம்.

கண்டுபிடிப்புகள்: 2016 மற்றும் 2018 க்கு இடையில், சிஎன்என்எஸ் கணக்கெடுப்பு 49,486 நபர்களிடமிருந்து இரத்த மாதிரிகளை சேகரித்தது. 41,210 பங்கேற்பாளர்கள் ஒரு ஹீமோகுளோபின் மதிப்பைக் கொண்டிருந்தனர், அவர்களில் 8087 பேர் எங்கள் ஆய்வில் சேர்க்கப்பட்டனர் மற்றும் முதன்மை பகுப்பாய்வு மாதிரியைக் கொண்டிருந்தனர். தற்போதுள்ள WHO மதிப்பீடு(cutoff)உடன் ஒப்பிடும்போது, ஹீமோகுளோபினுக்கான ஆய்வு மதிப்பீடு(Cutoff)எல்லா வயதிலும் குறைவாக இருந்தன, பொதுவாக 1-2 கிராம் / டி.எல்., ஆனால் 1–2 வயதுடைய குழந்தைகளிலும், 10 வயது அல்லது அதற்கு மேற்பட்ட

வயதுடைய சிறுமிகளிலும். அனைத்து வயதினருக்கும் பாலினத்துக்கும் செல்லுபடியாகும் ஹீமோகுளோபின் மதிப்புகள் கொண்ட முழு சி.என்.என்.எஸ் மாதிரியில் WHO மதிப்பீட்டை (cutoff) காட்டிலும் ஆய்வு மதிப்பீடு(cutoff)இரத்த சோகை பாதிப்பு 19 · 2 சதவீதம் குறைவாக இருந்தது (10·8% ஆய்வு மதிப்பீடு (cutoff), 30·0% WHO மதிப்பீடு.

விளக்கம்: இந்த கண்டுபிடிப்புகள் இரத்த சோகையை வரையறுக்க WHO ஹீமோகுளோபின் மதிப்பீடு (cutoff)மறுபரிசீலனை செய்வதற்கு துணைபுரிகின்றன. ஒரு பெரிய பிரதிநிதி இந்திய கணக்கெடுப்பில் ஆரோக்கியமான பங்கேற்பாளர்களிடமிருந்து பெறப்பட்ட எங்கள் ஹீமோகுளோபின் குறிப்பு சதவீதங்கள் இந்தியாவில் தேசிய பயன்பாட்டிற்கு ஏற்றவை. 1–19 வயது வரம்பில் மற்றும் பாலினங்களுக்கிடையில் ஹீமோகுளோபின் மதிப்புகளின் 5 வது சதவிகிதத்தில் கணிசமான வேறுபாடுகள் வசதிக்காக அடுக்கடுக்காக உள்ள வயதுக் குழுக்களில் பொதுவான மதிப்பீடு உருவாக்குவதற்கு எதிராக வாதிடுகின்றன.

நிதி: எதுவும் இல்லை
