# Supplementary material for: Haemoglobin thresholds to define anaemia in a national sample of healthy children and adolescents aged 1–19 years in India: a population-based study
Source: Lancet Glob Health. Author manuscript; Available in PMC 2022 Jul 5. (PMC7612991; doi:10.1016/S2214-109X(21)00077-2)
Supplement: Appendix 4 [file EMS146378-supplement-Appendix_4.pdf]

THE LANCET
Global Health

Supplementary appendix 4

This translation in Kannada was submitted by the authors and we reproduce it as supplied. It has not been peer reviewed. *The Lancet*'s editorial processes have only been applied to the original in English, which should serve as reference for this manuscript.

Supplement to: Sachdev HS, Porwal A, Acharya R, et al. Haemoglobin thresholds to define anaemia in a national sample of healthy children and adolescents aged 1–19 years in India: a population-based study. *Lancet Glob Health* 2021; published online April 16. https://doi.org/10.1016/S2214-109X(21)00077-2.

'[ಕನ್ನಡ ಭಾಷೆಯ] ಈ ಅನುವಾದವನ್ನು ಲೇಖಕರು ಸಲ್ಲಿಸಿದ್ದಾರೆ ಮತ್ತು ನಾವು ಅದನ್ನು ಸರಬರಾಜು ಮಾಡಲಾಗಿರುವಂತೆತೆ ಪುನರುತ್ಪಾದಿಸುತ್ತಿದ್ದೇವೆ. ಇದನ್ನು ಸಮಾನಸ್ಕಂದರು ಪರಿಶೀಲಿಸಿಲ್ಲ. ಲ್ಯಾನ್ಸೆಟ್‌ನ ಸಂಪಾದಕೀಯ ಪ್ರಕ್ರಿಯೆಗಳನ್ನು ಇಂಗ್ಲಿಷ್‌ನಲ್ಲಿನ ಮೂಲಕ್ಕೆ ಮಾತ್ರ ಅನ್ವಯಿಸಲಾಗಿದೆ, ಅದು ಈ ಹಸ್ತಪ್ರತಿಗೆ ಉಲ್ಲೇಖವಾಗಿ ಕಾರ್ಯನಿರ್ವಹಿಸುತ್ತದೆ. 'ಕಕ

ಭಾರತದ ೧-೧೯ ವರ್ಷ ವಯಸ್ಸಿನ ಆರೋಗ್ಯವಂತ ಮಕ್ಕಳು ಮತ್ತು ಹದಿಹರೆಯದವರ ರಾಷ್ಟ್ರೀಯ ಮಾದರಿಯ ಹಿಮೋಗ್ಲೋಬಿನ್ ಮತಿಗಳಿಂದ ರಕ್ತಹೀನತೆಯ ವ್ಯಾಖ್ಯಾನ: ಜನಸಂಖ್ಯೆ ಆಧಾರಿತ ಅಧ್ಯಯನ

ಸಾರಾಂಶ

<u>ಹಿನ್ನಲೆ</u>

ವಿಶ್ವ ಆರೋಗ್ಯ ಸಂಸ್ಥೆಯ ರಕ್ತಹೀನತೆಯ ಕಟ್‌ಆಫ್ ಪ್ರಧಾನವಾಗಿ ಐವತ್ತು ವರ್ಷಗಳ ಹಿಂದೆ ನಡೆಸಲಾದ ಶ್ವೇತವರ್ಣ ಜನಸಂಖ್ಯಾಯ ಐದು ಅಧ್ಯಯನಗಳ ಮೇಲೆ ಆಧಾರಿತವಾಗಿದೆ. ಆದ್ದರಿಂದ ಜಾಗತಿಕ ಪ್ರಯೋಗಕ್ಕೆ ಅನುಗುಣವಾಗಬೇಕಾದರೆ ರಕ್ತಹೀನತೆಯ ಕಟ್‌ಆಫ್‌ಗಳ ಪುನರ್ಪರಿಶೀಲನೆ ಆಯಾಯ ಜನಸಂಖ್ಯಿಯ ಆರೋಗ್ಯವಂತ ಮಕ್ಕಳು ಮತ್ತು ವಯಸ್ಕರಲ್ಲಿ ಮಾಡುವುದು ಅಗತ್ಯ. ಕಡಿಮೆ-ಆದಾಯ ಮತ್ತು ಮಧ್ಯಮ-ಆದಾಯ-ದೇಶಗಳಲ್ಲಿ ಇಂತಹ ಡೇಟಾ ವಿರಳವಾಗಿದೆ; ೨೦೧೯ ರಲ್ಲಿ ನಡೆದ ೦- ೧೯ ವರ್ಷ ವಯಸ್ಸಿನ ಮಕ್ಕಳು ಮತ್ತು ಹದಿಹರೆಯದವರ ದೊಡ್ಡ ಪ್ರಮಾಣದ ಭಾರತ ರಾಷ್ಟ್ರವನ್ನು ಪ್ರತಿನಿಧಿಸುವ ಸಮೀಕ್ಷೆಯಿಂದ (ಕಂಪ್ರೆಹೆನ್ಸಿವ್ ನ್ಯಾಷನಲ್ ನ್ಯೂಟ್ರಿಷನ್ ಸರ್ವೇ [ಸಿಎನ್‌ಎನ್‌ಎಸ್]) ಈ ಮರುಪರಿಶೀಲನೆಗೆ ಅವಕಾಶ ಸಿಕ್ಕಿತು. ಈ ಸಮೀಕ್ಷೆಯನ್ನು ಬಳಸಿಕೊಂಡು, ಸಿಎನ್‌ಎನ್‌ಎಸ್ ಜನಸಂಖ್ಯೆಯಲ್ಲಿ ವಯಸ್ಸು-ನಿರ್ದಿಷ್ಟ ಮತ್ತು ಲೈಂಗಿಕ-ನಿರ್ದಿಷ್ಟ ಹಿಮೋಗ್ಲೋಬಿನ್ ಶೇಕಡಾವಾರುಗಳನ್ನು ಮತ್ತು ರಕ್ತಹೀನತೆಯನ್ನು ಪತ್ತೆಹಚ್ಚುವ ಕಟ್‌ಆಫ್‌ಗಳನ್ನೂ ನಿರ್ಣಯಿಸಲು ನಾವು ಗುರಿ ಹೊಂದಿದ್ದೇವೆ.

<u>ವಿಧಾನಗಳು</u>

ಈ ಜನಸಂಖ್ಯೆ ಆಧಾರಿತ ಅಧ್ಯಯನಕ್ಕಾಗಿ, ಸಿಎನ್‌ಎನ್‌ಎಸ್‌ನಲ್ಲಿ ವ್ಯಾಖ್ಯಾನಿಸಲಾದ ಆರೋಗ್ಯಕರ ಜನಸಂಖ್ಯೆಗಾಗಿ ವರದಿ ಮಾಡಲಾದ ಮೌಲ್ಯಗಳಿಂದ ವಯಸ್ಸು-ನಿರ್ದಿಷ್ಟ ಮತ್ತು ಲೈಂಗಿಕ-ನಿರ್ದಿಷ್ಟ ಹಿಮೋಗ್ಲೋಬಿನ್ ಶೇಕಡಾವಾರುಗಳನ್ನು ನಾವು ನಿರ್ಮಿಸಿದ್ದೇವೆ, ಈ ಸಮೀಕ್ಷೆಯು, ಮಾದರಿ ಸಂಗ್ರಹದ ಸಮಯದಲ್ಲಿ ಮತ್ತು ಪ್ರಯೋಗಾಲಯದ ವಿಶ್ಲೇಷಣೆಗಳಲ್ಲಿ ಕಠಿಣ ಗುಣಮಟ್ಟದ ನಿಯಂತ್ರಣ ಕ್ರಮಗಳನ್ನು ಬಳಸಿದೆ. ಆರೋಗ್ಯಕರ ಜನಸಂಖ್ಯೆಯನ್ನು ಪಡೆಯಲು, ನಾವು ಕಬ್ಬಿಣ, ಫೋಲೇಟ್, ವಿಟಮಿನ್ ಬಿ$_{೧೨}$ ಮತ್ತು ರೆಟಿನಾಲ್ ಕೊರತೆ ಹೊಂದಿರುವವರನ್ನು ಪರಿಗಣಿಸಲಿಲ್ಲ; ಉರಿಯೂತ, ಭಿನ್ನ ಹಿಮೋಗ್ಲೋಬಿನ್ಗಳು (ಹಿಮೋಗ್ಲೋಬಿನ್ ಎ$_{೨}$ ಮತ್ತು ಹಿಮೋಗ್ಲೋಬಿನ್ ಎಸ್), ಮತ್ತು ಧೂಮಪಾನದ ಇತಿಹಾಸವಿರುವವರನ್ನು ಪರಿಗಣಿಸಲಿಲ್ಲ. ಈ ಅಧ್ಯಯನದಲ್ಲಿ ನಾವು ರಕ್ತಹೀನತೆಯನ್ನು ವ್ಯಾಖ್ಯಾನಿಸಲು ಆರೋಗ್ಯಕರ ಜನಸಂಖ್ಯೆಗಾಗಿ ಪಡೆದ ಹಿಮೋಗ್ಲೋಬಿನ್‌ನ ವಯಸ್ಸು-ನಿರ್ದಿಷ್ಟ ಮತ್ತು ಲೈಂಗಿಕ-ನಿರ್ದಿಷ್ಟ ೫ನೇ ಶೇಕಡಾವಾರುವನ್ನು ಪರಿಗಣಿಸಿದ್ದೇವೆ. ಇಡೀ ಸಿಎನ್‌ಎನ್‌ಎಸ್ ಮಾದರಿಯಲ್ಲಿ ರಕ್ತಹೀನತೆ ಇರುವ ಜನಸಂಖ್ಯಿಯ ಪ್ರಮಾಣವನ್ನು ಪ್ರತಿ ವಯಸ್ಸಿನಲ್ಲಿ ಮತ್ತು ಲೈಂಗಿಕತೆಯ ನಡುವಿನ ಗಮನಾರ್ಹ ವ್ಯತ್ಯಾಸಗಳನ್ನು ನಿರ್ಣಯಿಸಲು ನಾವು ಅಸ್ತಿತ್ವದಲ್ಲಿರುವ ವಿಶ್ವ ಆರೋಗ್ಯ ಸಂಸ್ಥೆಯ ಕಟ್‌ಆಫ್‌ಗಳೊಂದಿಗೆ ಹೋಲಿಸಿದ್ದೇವೆ.

<u>ಫಲಿತಾಂಶಗಳು</u>

೨೦೧೬ ಮತ್ತು ೨೦೧೮ ರ ನಡುವೆ, ಸಿಎನ್‌ಎನ್‌ಎಸ್ ಸಮೀಕ್ಷೆಯು ೪೯,೪೮೬ ಜನರಿಂದ ರಕ್ತದ ಮಾದರಿಗಳನ್ನು ಸಂಗ್ರಹಿಸಿದ್ದು, ಅದರಲ್ಲಿ, ೪೧,೨೧೦ ಭಾಗಿಗಳು ಹಿಮೋಗ್ಲೋಬಿನ್ ಮೌಲ್ಯವನ್ನು ಹೊಂದಿದ್ದರು, ಅವರಲ್ಲಿ ೮೦೮೭

ಜನರನ್ನು ನಮ್ಮ ಅಧ್ಯಯನದಲ್ಲಿ ಸೇರಿಸಕೊಳ್ಳಲಾಗಿತ್ತು ಮತ್ತು ಅವರು ಪ್ರಾಥಮಿಕ ವಿಶ್ಲೇಷಣಾತ್ಮಕ ಮಾದರಿಗೂ ಒಳಗೊಂಡಿದ್ದರು. ಅಸ್ತಿತ್ವದಲ್ಲಿರುವ ವಿಶ್ವ ಆರೋಗ್ಯ ಸಂಸ್ಥೆಯ ಕಟ್‌ಆಫ್‌ಗಳಿಗೆ ಹೋಲಿಸಿದರೆ, ಹಿಮೋಗ್ಲೋಬಿನ್‌ನ ಈ ಅಧ್ಯಯನದ ಹಿಮೋಗ್ಲೋಬಿನ್ ಕಟ್‌ಆಫ್‌ಗಳು ಎಲ್ಲಾ ವಯಸ್ಸಿನಲ್ಲೂ ಸಾಮಾನ್ಯವಾಗಿ ೧-೨ ಗ್ರಾಂ / ಡಿಎಲ್ ನಿಂದ ಕಡಿಮೆಯಾಗಿದ್ದವು, ಆದರೆ ಎರಡೂ ಲಿಂಗಗಳ ೧-೨ ವರ್ಷ ವಯಸ್ಸಿನ ಮತ್ತು ೧೦ ವರ್ಷ ಅಥವಾ ಅದಕ್ಕಿಂತ ಹೆಚ್ಚಿನ ವಯಸ್ಸಿನ ಹುಡುಗಿಯರಲ್ಲಿ ಇದು ಹೆಚ್ಚಾಗಿತು. ನಮ್ಮ ಅಧ್ಯಯನದ ಕಟ್‌ಆಫ್ ಪ್ರಕಾರ ಇಡೀ ಸಿಎನ್‌ಎನ್‌ಎಸ್ ಮಾದರಿಯಲ್ಲಿ ಎಲ್ಲಾ ವಯಸ್ಸಿನ ಮತ್ತು ಲಿಂಗಗಳಾದ್ಯಂತ ಹಿಮೋಗ್ಲೋಬಿನ್ ಮೌಲ್ಯಗಳೊಂದಿಗೆ (೧೦.೮% ಈ ಅಧ್ಯಯನದ ಕಟ್‌ಆಫ್‌ಗಳು ಮತ್ತು ೩೦.೦% ವಿಶ್ವ ಆರೋಗ್ಯ ಸಂಸ್ಥೆಯ ಕಟ್‌ಆಫ್) ರಕ್ತಹೀನತೆ ಇರುವ ಜನಸಂಖ್ಯೆಯ ಪ್ರಮಾಣ ವಿಶ್ವ ಆರೋಗ್ಯ ಸಂಸ್ಥೆಯ ಕಟ್‌ಆಫ್‌ಗಳಿಗಿಂತ ೧೯. ೨ ಶೇಕಡಾ ಕಡಿಮೆಯಾಗಿದೆ.

ವ್ಯಾಖ್ಯಾನ

ರಕ್ತಹೀನತೆಯನ್ನು ವ್ಯಾಖ್ಯಾನಿಸಲು ವಿಶ್ವ ಆರೋಗ್ಯ ಸಂಸ್ಥೆಯ ಹಿಮೋಗ್ಲೋಬಿನ್ ಕಟ್‌ಆಫ್‌ಗಳ ಮರು ಪರೀಕ್ಷೆಯನ್ನು ಈ ಸಂಶೋಧನೆ ಬೆಂಬಲಿಸುತದೆ. ಈ ಸಂಶೋಧನೆಯ ಹಿಮೋಗ್ಲೋಬಿನ್ ಶೇಕಡಾವಾರು, ಭಾರತೀಯ ಆರೋಗ್ಯವಂತ ಜನರ ದೊಡ್ಡ ಪ್ರಮಾಣದ ಸಮೀಕ್ಷೆಯಿಂದ ಪಡೆಯಲಾಗಿದೆ. ಆದ್ದರಿಂದ ಇದು ಭಾರತದಲ್ಲಿ ರಾಷ್ಟ್ರೀಯ ಬಳಕೆಗೆ ಸೂಕ್ತವಾಗಿದೆ. ೧-೧೯ ವರ್ಷ ವಯಸ್ಸಿನ ವ್ಯಾಪ್ತಿಯಲ್ಲಿ ಮತ್ತು ಲಿಂಗಗಳ ನಡುವಿನ ೫ನೇ ಶೇಕಡಾವಾರು ಹಿಮೋಗ್ಲೋಬಿನ್ ಮೌಲ್ಯಗಳು ಗಣನಾರ್ಹ ವ್ಯತ್ಯಾಸ ಇರುವರಿಂದ, ಅನುಕೂಲಕ್ಕಾಗಿ ಶ್ರೇಣೀಕೃತ ವಯಸ್ಸಿನ ಗುಂಪುಗಳಲ್ಲಿ ಸಾಮಾನ್ಯ ಕಟ್‌ಆಫ್‌ಗಳನ್ನು ನಿರ್ಮಿಸುವುದರ ವಿರುದ್ಧ ವಾದಿಸುತೇವೆ.

ಧನಸಹಾಯ: ಯಾವುದೂ ಇಲ್ಲ
