# Supplementary material for: Haemoglobin thresholds to define anaemia in a national sample of healthy children and adolescents aged 1–19 years in India: a population-based study
Source: Lancet Glob Health. Author manuscript; Available in PMC 2022 Jul 5. (PMC7612991; doi:10.1016/S2214-109X(21)00077-2)

THE LANCET
Global Health

Supplementary appendix 5

This appendix formed part of the original submission and has been peer reviewed. We post it as supplied by the authors.

Supplement to: Sachdev HS, Porwal A, Acharya R, et al. Haemoglobin thresholds to define anaemia in a national sample of healthy children and adolescents aged 1–19 years in India: a population-based study. *Lancet Glob Health* 2021; published online April 16. https://doi.org/10.1016/S2214-109X(21)00077-2.

Online Supplementary Material

Haemoglobin thresholds to define anaemia in a national sample of healthy children and adolescents aged 1–19 years in India: a population-based study

Harshpal Singh Sachdev, Akash Porwal, Rajib Acharya, Sana Ashraf, Sowmya Ramesh, Nizamuddin Khan, Umesh Kapil, Anura V Kurpad, Avina Sarna

Supplementary Table 1: Socio-demographic characteristics of the primary analytic sample and their comparison with the non-analytic sample.

Background characteristics	1-4 years					5-9 years					10-19 years				
	Non-Analytical Sample$		Analytical Sample#			Non-Analytical Sample$		Analytical Sample#			Non-Analytical Sample$		Analytical Sample#		
	n	% /Mean (SD)	n	% /Mean (SD)	p-value	n	% /Mean (SD)	n	% /Mean (SD)	p-value	n	% /Mean (SD)	n	% /Mean (SD)	p-value
**Age (in years)**	9479	2.7 (1.1)	2137	2.9 (1.0)	<0.001	11670	7.0 (1.4)	3461	7.1 (1.4)	0.556	11963	14.3 (2.8)	2489	13.9 (2.8)	<0.001
**Hemoglobin (in g/dl)**	9479	11.4 (1.7)	2137	11.9 (1.1)	<0.001	11670	12.3 (1.4)	3461	12.5 (1.0)	<0.001	11963	12.9 (1.8)	2489	13.1 (1.3)	<0.001
**Anthropometric Measures**															
HAZ (z-score)	9225	-1.4 (1.4)	2067	-1.2 (1.3)	<0.001	11247	-1.0 (1.3)	3351	-0.8 (1.2)	<0.001	10980	-1.3 (1.1)	2298	-1.2 (1.2)	0.035
WAZ (z-score)	9263	-1.3 (1.2)	2069	-1.2 (1.2)	<0.001	11442	-1.3 (1.3)	3350	-1.1 (1.3)	<0.001	NA	NA	NA	NA	NA
WHZ (z-score)	9084	-0.8 (1.2)	2032	-0.8 (1.2)	0.025	NA	NA	NA	NA	NA	NA	NA	NA	NA	NA
BMI-for-age (z-score)	NA	NA	NA	NA	NA	11334	-1.0 (1.2)	3323	-0.9 (1.3)	<0.001	10947	-0.9 (1.3)	2287	-0.9 (1.32)	0.676
**Sex**					0.083					0.003					
Boys	5098	53.8	1105	51.7		6105	52.3	1910	55.2		6019	50.3	1360	54.6	
Girls	4381	46.2	1032	48.3		5565	47.7	1551	44.8		5944	49.7	1129	45.4	
**Place of Residence**					0.158					0.002					0.001
Rural	5362	56.6	1173	54.9		6738	57.7	1895	54.8		6953	58.1	1356	54.5	
Urban	4117	43.4	964	45.1		4932	42.3	1566	45.3		5010	41.9	1133	45.5	
**Region**					<0.001					<0.001					<0.001
North	1860	19.6	407	19.1		2541	21.8	795	23.0		2720	22.7	628	25.2	
Central	793	8.4	104	4.9		1327	11.4	219	6.3		1305	10.9	132	5.3	
East	1743	18.4	576	27.0		1888	16.2	839	24.2		1891	15.8	626	25.2	
West	1219	12.9	118	5.5		1450	12.4	203	5.9		1467	12.3	140	5.6	
South	1608	17.0	338	15.8		1839	15.8	482	13.9		1950	16.3	260	10.5	
North-East	2256	23.8	594	27.8		2525	22.5	923	26.7		2630	22.0	703	28.2	
**Religion**					<0.001					<0.001					<0.001
Hindu	6780	71.5	1486	69.5		8461	72.5	2410	69.6		8850	74.0	1692	68.0	
Muslim	1085	11.5	314	14.7		1273	10.9	524	15.1		1240	10.4	387	15.6	
Christian	992	10.5	222	10.4		1235	10.6	327	9.5		1172	9.8	264	10.6	
Sikh	236	2.5	21	1.0		273	2.3	48	1.4		300	2.5	35	1.4	

Background characteristics	1-4 years					5-9 years					10-19 years				
	Non-Analytical Sample[$]		Analytical Sample[#]			Non-Analytical Sample[$]		Analytical Sample[#]			Non-Analytical Sample[$]		Analytical Sample[#]		
	n	% /Mean (SD)	n	% /Mean (SD)	p-value	n	% /Mean (SD)	n	% /Mean (SD)	p-value	n	% /Mean (SD)	n	% /Mean (SD)	p-value
Others	386	4.1	94	4.4		428	3.7	152	4.4		401	3.4	111	4.5	
**Wealth Index**					<0.001					<0.001					<0.001
Poorest	724	7.6	121	5.7		903	7.7	186	5.4		1017	8.5	165	6.6	
Poor	1237	13.1	202	9.5		1423	12.2	364	10.5		1632	13.6	298	12.0	
Middle	2013	21.2	418	19.56		2279	19.5	648	18.7		2398	20.1	471	18.9	
Rich	2601	27.4	626	29.3		3285	28.2	969	28.0		3159	26.4	691	27.8	
Richest	2904	30.6	770	36.0		3780	32.4	1294	37.4		3757	31.4	864	34.7	

[$]Non-Analytical Sample: Overall participants with a blood sample collected, excluding participants in analytical sample 1 (see below).

[#]Analytical Sample 1 (See text): Excluded participants with iron, folate, vitamin B12 and retinol deficiencies, inflammation (based on C-reactive protein), variant Hb (HbA2 and HbS), and smokers.

NA: Not Applicable

Supplementary Table 2. Smoothed haemoglobin (g/dl) percentiles from primary analytic sample at yearly intervals from 1-19 years.

	Boys										Girls									
**Age (years)**	**M**	**S**	**L**	**C5**	**C25**	**C50**	**C75**	**C85**	**C90**	**C95**	**M**	**S**	**L**	**C5**	**C25**	**C50**	**C75**	**C85**	**C90**	**C95**
**1**	11.03	0.12	-0.10	9.12	10.20	11.03	11.94	12.46	12.83	13.39	10.97	0.12	-1.38	9.25	10.18	10.97	11.93	12.53	12.98	13.73
**2**	11.28	0.11	-0.10	9.46	10.49	11.28	12.14	12.63	12.97	13.50	11.26	0.11	-1.25	9.60	10.51	11.26	12.16	12.71	13.11	13.77
**3**	11.52	0.10	-0.10	9.77	10.77	11.52	12.33	12.79	13.12	13.61	11.54	0.10	-1.12	9.92	10.81	11.54	12.37	12.88	13.25	13.84
**4**	11.74	0.09	-0.09	10.06	11.02	11.74	12.52	12.96	13.26	13.73	11.77	0.09	-0.99	10.19	11.07	11.77	12.57	13.04	13.39	13.93
**5**	11.93	0.09	-0.09	10.30	11.23	11.93	12.68	13.10	13.40	13.85	11.96	0.09	-0.86	10.40	11.27	11.96	12.73	13.19	13.51	14.02
**6**	12.09	0.09	-0.09	10.49	11.40	12.09	12.81	13.22	13.51	13.95	12.10	0.09	-0.73	10.54	11.42	12.10	12.87	13.31	13.63	14.12
**7**	12.22	0.08	-0.09	10.65	11.55	12.22	12.93	13.33	13.61	14.04	12.21	0.09	-0.59	10.62	11.52	12.21	12.98	13.42	13.73	14.22
**8**	12.33	0.08	-0.08	10.78	11.67	12.33	13.04	13.43	13.71	14.13	12.30	0.09	-0.46	10.68	11.59	12.30	13.06	13.51	13.82	14.30
**9**	12.44	0.08	-0.08	10.88	11.77	12.44	13.15	13.55	13.83	14.25	12.36	0.09	-0.33	10.71	11.65	12.36	13.13	13.57	13.88	14.36
**10**	12.56	0.08	-0.08	10.97	11.88	12.56	13.29	13.70	13.98	14.41	12.40	0.09	-0.20	10.73	11.68	12.40	13.17	13.60	13.91	14.38
**11**	12.70	0.08	-0.08	11.06	12.00	12.70	13.45	13.87	14.17	14.61	12.40	0.09	-0.07	10.73	11.69	12.40	13.16	13.59	13.89	14.35
**12**	12.87	0.09	-0.07	11.17	12.14	12.87	13.64	14.07	14.38	14.84	12.38	0.09	0.07	10.72	11.68	12.38	13.13	13.55	13.84	14.29
**13**	13.05	0.09	-0.07	11.32	12.31	13.05	13.84	14.29	14.60	15.07	12.36	0.09	0.20	10.70	11.66	12.36	13.10	13.51	13.79	14.22
**14**	13.26	0.09	-0.07	11.50	12.50	13.26	14.06	14.51	14.82	15.30	12.34	0.09	0.33	10.68	11.64	12.34	13.06	13.46	13.74	14.16
**15**	13.48	0.09	-0.07	11.71	12.72	13.48	14.28	14.74	15.05	15.53	12.30	0.09	0.46	10.64	11.60	12.30	13.02	13.42	13.69	14.10
**16**	13.71	0.08	-0.06	11.94	12.95	13.71	14.51	14.97	15.28	15.76	12.26	0.09	0.59	10.59	11.56	12.26	12.98	13.37	13.64	14.04
**17**	13.94	0.08	-0.06	12.18	13.19	13.94	14.74	15.19	15.50	15.97	12.23	0.09	0.73	10.54	11.53	12.23	12.95	13.34	13.60	14.00
**18**	14.16	0.08	-0.06	12.42	13.42	14.16	14.95	15.40	15.71	16.17	12.23	0.09	0.86	10.51	11.52	12.23	12.94	13.33	13.59	13.98
**19**	14.38	0.08	-0.06	12.65	13.64	14.38	15.16	15.60	15.90	16.36	12.24	0.09	0.99	10.49	11.52	12.24	12.96	13.35	13.61	14.00

C: Centile; for example, C5 refers to 5th centile (percentile); L: Skewness of distribution; M: Median of distribution; S: Coefficient of variation of distribution

Supplementary Figure 1. Comparison of mean and their 95% CIs (bands) of haemoglobin in the five analytic samples.

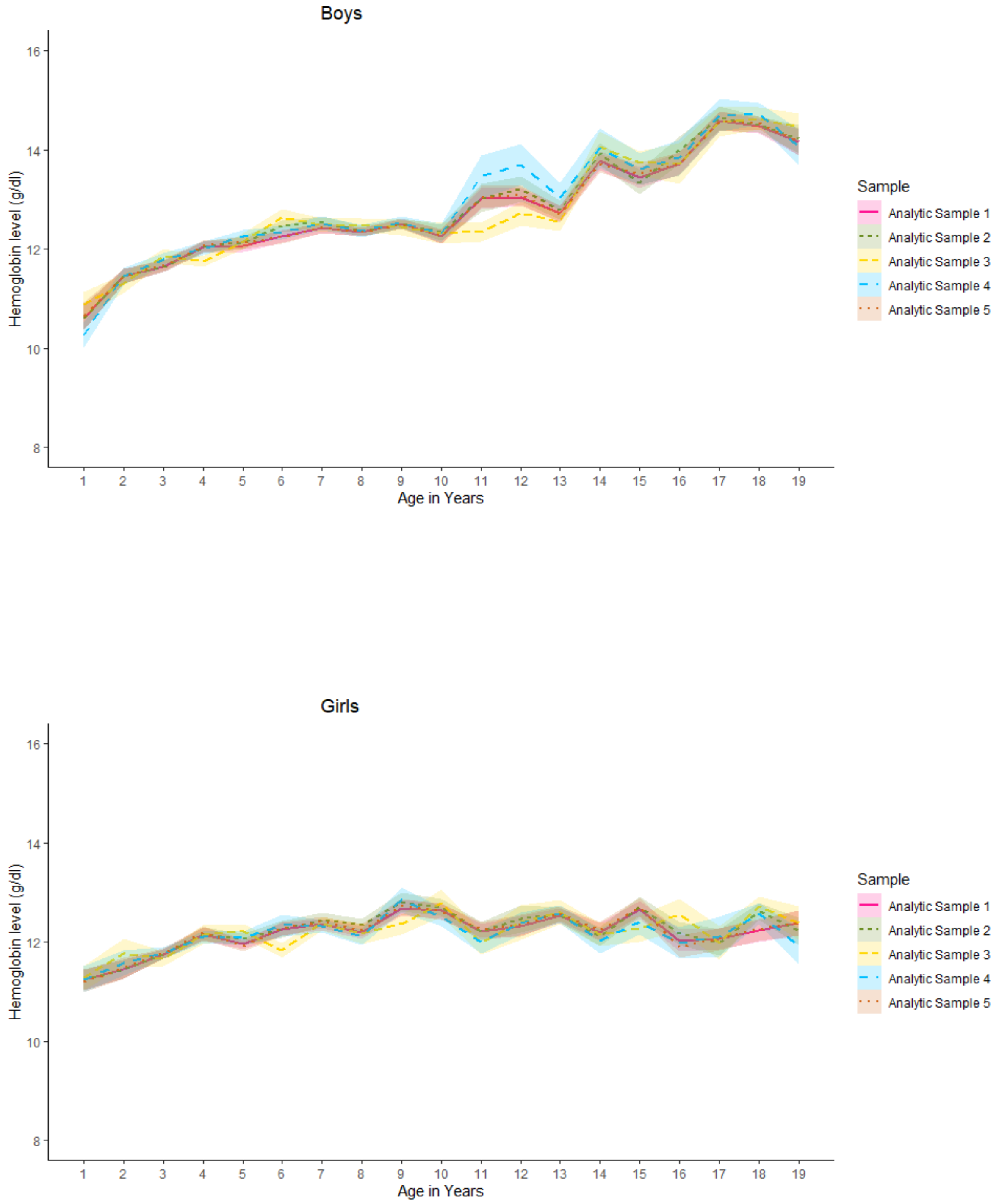

Supplementary Figure 2. Comparison of 5th percentile and their 95% CIs (bands) of haemoglobin in the five analytic samples.

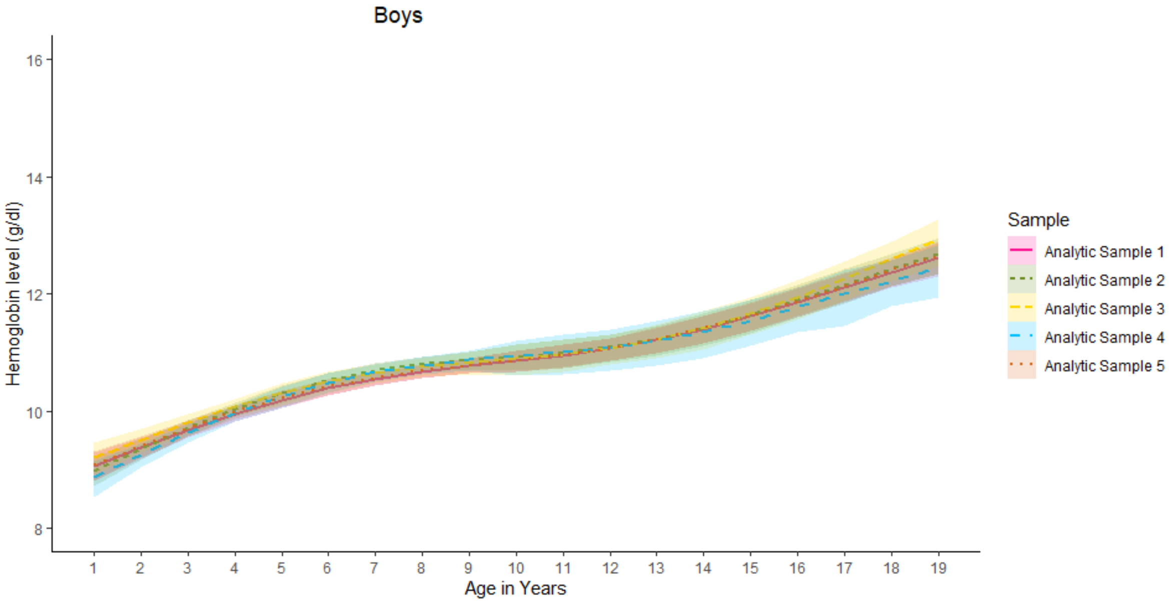

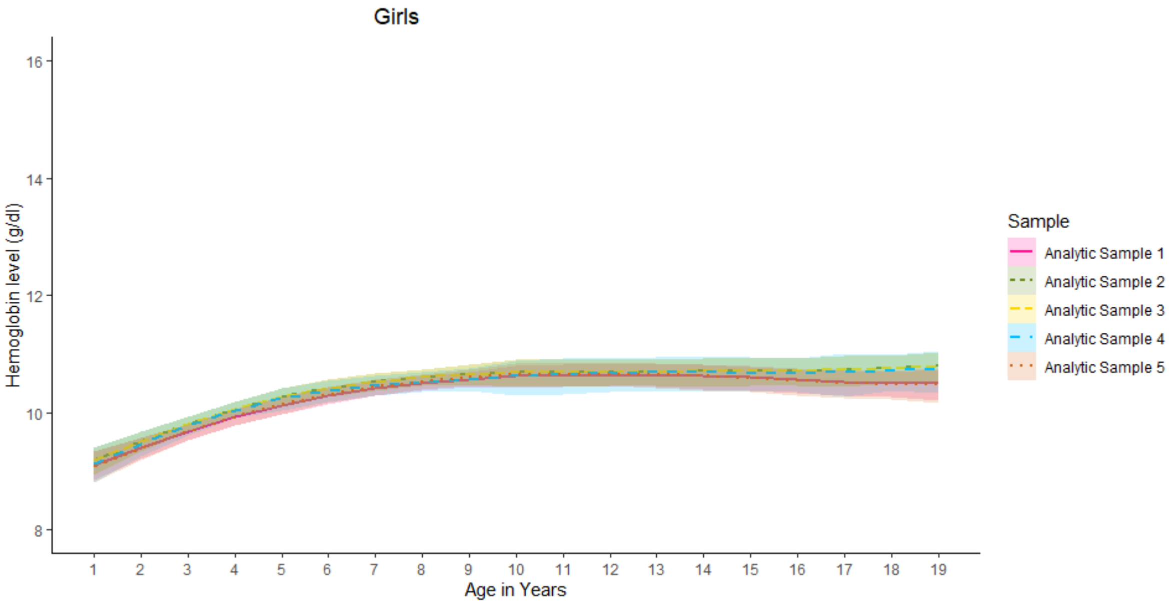

Supplement: Appendix 5 [file EMS146378-supplement-Appendix_5.pdf]
